# Structure of the respiratory MBS complex reveals iron-sulfur cluster catalyzed sulfane sulfur reduction in ancient life

**DOI:** 10.1038/s41467-020-19697-7

**Published:** 2020-11-23

**Authors:** Hongjun Yu, Dominik K. Haja, Gerrit J. Schut, Chang-Hao Wu, Xing Meng, Gongpu Zhao, Huilin Li, Michael W. W. Adams

**Affiliations:** 1 Structural Biology Program, Van Andel Institute, Grand Rapids, MI USA; 2 Department of Biochemistry and Molecular Biology, University of Georgia, Athens, GA USA; 3 Present Address: Department of Biochemistry and Molecular Biology, School of Basic Medicine and Tongji Medical College, Huazhong University of Science and Technology, Wuhan, China

**Keywords:** Metalloproteins, Cryoelectron microscopy

## Abstract

Modern day aerobic respiration in mitochondria involving complex I converts redox energy into chemical energy and likely evolved from a simple anaerobic system now represented by hydrogen gas-evolving hydrogenase (MBH) where protons are the terminal electron acceptor. Here we present the cryo-EM structure of an early ancestor in the evolution of complex I, the elemental sulfur (S^0^)-reducing reductase MBS. Three highly conserved protein loops linking cytoplasmic and membrane domains enable scalable energy conversion in all three complexes. MBS contains two proton pumps compared to one in MBH and likely conserves twice the energy. The structure also reveals evolutionary adaptations of MBH that enabled S^0^ reduction by MBS catalyzed by a site-differentiated iron-sulfur cluster without participation of protons or amino acid residues. This is the simplest mechanism proposed for reduction of inorganic or organic disulfides. It is of fundamental significance in the iron and sulfur-rich volcanic environments of early earth and possibly the origin of life. MBS provides a new perspective on the evolution of modern-day respiratory complexes and of catalysis by biological iron-sulfur clusters.

## Abstract (web summary)

The sulfur-reducing enzyme MBS and the hydrogen-gas evolving MBH are the evolutionary link between the ancestor Mrp antiporter and the mitochondrial respiratory complex I. Here, the authors characterise MBS from the hyperthermophilic archaeon *Pyrococcus furiosus*, solve its cryo-EM structure and discuss the structural evolution from Mrp to MBH and MBS and to the modern-day complex I.

## Introduction

**Fig. 1:**
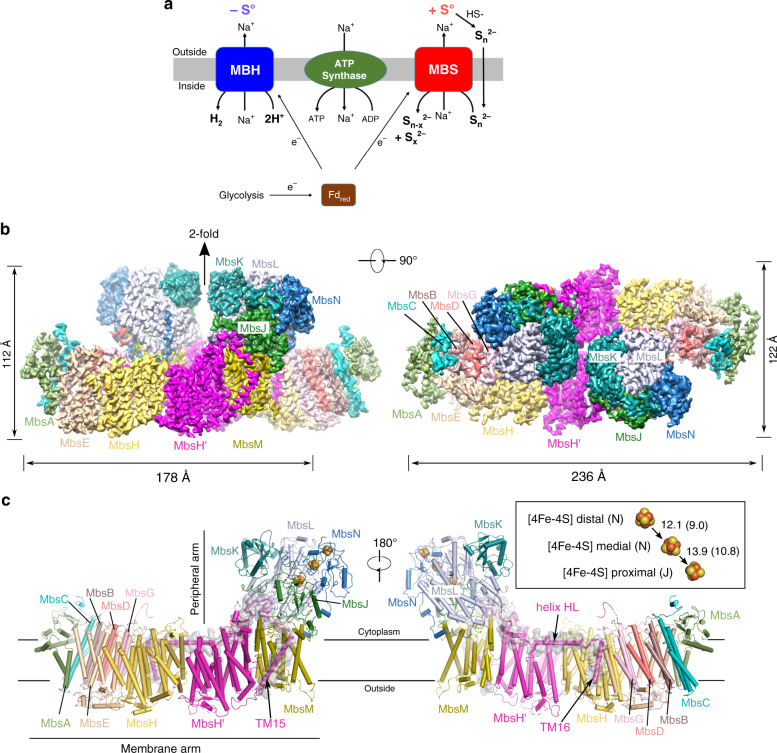
Overall structure of the *P. furiosus* MBS complex. **a** Schematic comparison of *P. furiosus* MBH and MBS complexes, both generating a Na^+^ gradient for ATP synthesis. **b** Side (left) and top (right) views of the cryo-EM map of the dimeric MBS complex, segmented and colored by subunits. **c** The front (left) and back (right) view of the atomic model of an MBS monomeric complex. Individual subunits are colored as in (**b**). Inset in the right panel: a chain of three [4Fe-4S] clusters in the peripheral arm, with the center-to-center and edge-to-edge distances (in brackets) between metal sites indicated in Å.

Respiratory complexes couple the energy released from spontaneous electron transfer reactions to the generation of chemical gradients. From archaea and bacteria to mammalian mitochondria, modern-day respiratory complex I contains a 14-subunit core^1–6^ that oxidizes NADH, reduces quinone, and translocates four protons across the membrane, used to drive ATP synthesis thereby conserving energy^7–11^. Such complexes first appeared billions of years ago in an anaerobic environment where, without oxygen, oxidative microbial metabolism ultimately reduced the ubiquitous proton to produce hydrogen gas catalyzed by a membrane-bound [NiFe]-hydrogenase (Fig. 1a, MBH)^6,12–15^. As the atmosphere became more oxidized, ancestral MBH evolved to reduce elemental sulfur (S^0^), now represented by sulfane sulfur reductase or MBS, and ultimately to reducing quinone, as in complex I^12–15^ and related NDH^16^.

MBH and MBS are found in the anaerobic hyperthermophilic archaeon *Pyrococcus furiosus*^17^, which oxidizes the redox protein ferredoxin, produces H_2_S or H_2_, and conserves energy by pumping Na^+^ (Fig. 1a)^9,12,18,19^. In the presence of S^0^, the genes encoding MBS are expressed while those encoding MBH are repressed^19–21^, resulting in a microbial cell yield twice that when cells are grown without S^0^^9,22^, suggesting that MBS is more efficient in energy conservation although the underlying mechanism is unknown. MBS reductively cleaves linear polysulfide chains (S_n_^2−^, where *n* ≥ 4), produced abiotically from the reaction of H_2_S and S^0^ (Eq. 1) in the hydrothermal vent environments that *P. furiosus* inhabits, into two shorter polysulfides (Eq. 2). Further reduction yields unstable di- and trisulfides (Eq. 3) that spontaneously convert to S^0^ and H_2_S (Eq. 4). S–S bond cleavage by MBS does not involve protons (Eqs. 2 and 3) and H_2_S is produced abiotically (Eq. 4). Bacterial polysulfide reductase (PSR, not present in *P. furiosus*) uses a molybdopterin cofactor to reductively cleave the terminal sulfur of polysulfides as H_2_S (Eq. 5)^23,24^ so the archaeal MBS must reduce a disulfide bond by a different mechanism.

1 $${{{\mathrm{Abiotic:S}}}}_8 + {{{\mathrm{H}}}}_{_{{{\mathrm{2}}}}}{{{\mathrm{S}}}} \to ^ - {{{\mathrm{S - S - S - S - S - S - S - S - S}}}}^{{{\mathrm{ - }}}}\left( {{{{\mathrm{S}}}}_{_9}^{2 - }} \right) + 2\;{{{\mathrm{H}}}}^{^ + }$$

2 $${{{\mathrm{MBS:S}}}}_9^{2 - } + 2\;e^ - \to ^ - {{{\mathrm{S - S - S - S}}}}^{{{\mathrm{ - }}}}{{{\mathrm{ + }}}}^{{{\mathrm{ - }}}}{{{\mathrm{S - S - S - S - S}}}}^{{{\mathrm{ - }}}}\left( {{{{\mathrm{S}}}}_{_5}^{2 - }} \right)$$

3 $${{{\mathrm{MBS:S}}}}_{_5}^{2 - } + 2\;e^ - \to ^ - {{{\mathrm{S - S - S}}}}^{{{\mathrm{ - }}}}{{{\mathrm{ + }}}}^{{{\mathrm{ - }}}}{{{\mathrm{S - S}}}}^{{{\mathrm{ - }}}}\left( {{{{\mathrm{S}}}}_2^{2 - }} \right)$$

4 $${{{\mathrm{Abiotic:}}}}\ 5\;{{{\mathrm{S}}}}_{_2}^{2 - } + 4\;{{{\mathrm{H}}}}^ + \to {{{\mathrm{S}}}}_8 + 2 \ {{{\mathrm{ H}}}}_2{{{\mathrm{S}}}}$$

5 $${{{\mathrm{PSR:S}}}}_{{{\mathrm{n}}}}^{2 - } + 2\;{{{\mathrm{e}}}}^ - + 2 \ {{{\mathrm{H}}}}^ + \to {{{\mathrm{S}}}}_{{{{\mathrm{n - 1}}}}}^{^{2 - }} + {{{\mathrm{H}}}}_2{{{\mathrm{S}}}}$$

The structure of MBH (298 kDa, 14 subunits) revealed its close evolutionary relationship with complex I. Although MBH contains a single proton pump compared to four in complex I, they have the same structural elements thought to enable transduction of redox energy to an ion gradient^6,7,25–27^. Herein we describe the structure of MBS (357 kDa, 13 subunits), the proposed evolutionary link between them^9^, and how a catalytic site evolved from reducing protons to reducing large hydrophobic molecules like S_n_^2–^ and cleaving an inorganic sulfur–sulfur bond, a reaction of high significance in the primordial early earth.

## Results

### Structure of MBS

**Fig. 2:**
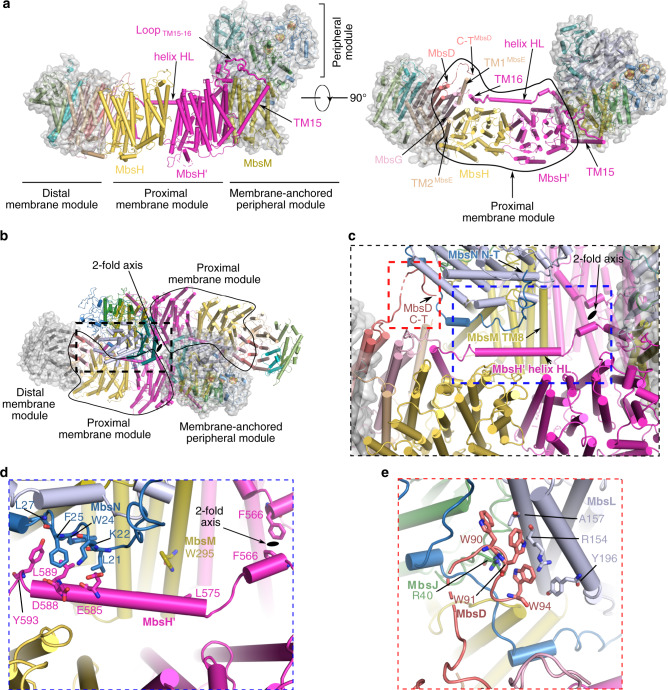
The architecture of the dimeric MBS complex. **a** Side (left) and top cytoplasmic views (right) of an MBS monomer. The central proximal membrane module is flanked by the distal membrane module and the membrane-anchored peripheral module, which are further highlighted by transparent surface views. **b** A top view showing the assembly of the dimeric MBS complex with the three different modules indicated as in (**a**). **c** A zoomed view of the dashed rectangle in (**b**). The dimer interface is composed of two regions, one involving the Helix HL of MbsH’ (dashed blue box) and the other involving the elongated C-terminal tail of MbsD (dashed red box). **d** A close-up view of the dashed blue box in (**c**). **e** A close-up view of the dashed red box in (**c**). Residues contributing to the dimer interface are shown as sticks in (**d**) and (**e**).

We determined the cryo-EM structure of MBS in a dimer complex form (Fig. 1b, c, Supplementary Fig. 1, Supplementary Fig. 2, Supplementary Table 1). The atomic model contained 26 protein subunits and 116 transmembrane helices (TMHs). The MBS monomer consists of a peripheral cytoplasmic arm (MbsJ, K, L, and N), anchored to the membrane by MbsM, and coordinates three [4Fe-4S] clusters that transfer electrons from ferredoxin to the substrate polysulfide (Fig. 1c)^19^. The membrane arm (MbsA, B, C, D, E, G, H, H’ and M) has 58 TMHs and contains a module proximal to the peripheral arm (proximal membrane module; MbsH’, MbsH, MbsD, MbsG and TMH1-2 of MbsE) and a distal membrane module (MbsA, B and C, and TMH3-6 of MbsE) (Fig. 2a, Supplementary Fig. 3a).

The MBS dimer complex is assembled via extensive contacts at two main places between the proximal membrane module of one MBS and the membrane-anchored peripheral module of the other (Figs. 1b, 2a–c). The first contact involves a long amphipathic horizontal helix (termed helix HL) of MbsH’ (Fig. 2d): the MbsH’ helix HL of one MBS interacts extensively with the N-terminal region of MbsN and TMH8 of MbsM in the other MBS, including several hydrophobic interactions and a salt bridge. The twofold axis is located between two F566 residues preceding the helix HL in the two MbsH’ of the MBS dimer complex. The second contact involves the tryptophan-rich C-terminal extension (W90, W91, and W94) of MbsD of one MBS projecting into a largely hydrophobic cleft in the peripheral domain of the other (Fig. 2e). The distal membrane module is not involved in the MBS dimer interface and is likely somewhat dynamic, which may explain the lower local resolution in this region (Supplementary Fig. 1g).

### MbsH and MbsH’ each contain a proton pump

**Fig. 3:**
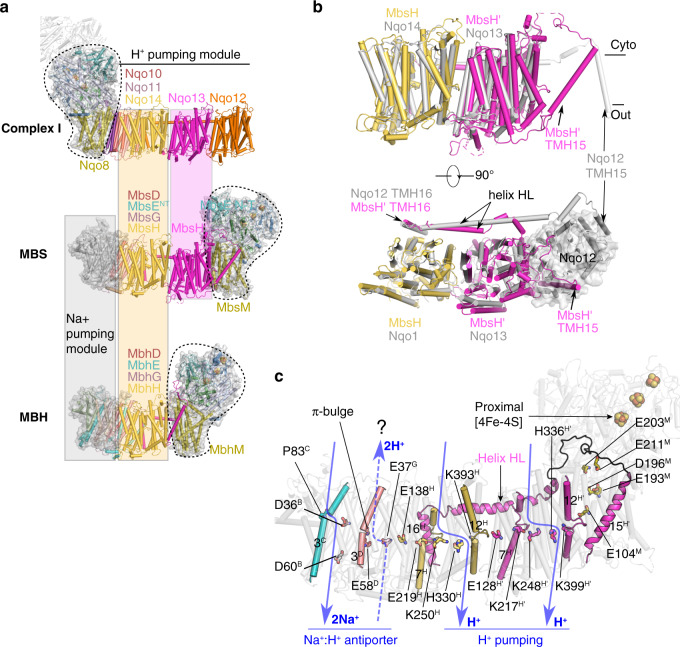
Assembly of the modular architecture of MBS and comparison with complex I and MBH. **a** Complex I (*T. thermophilus*; PDB ID 4HEA) is lined up with MBS and MBH (*P. furiosus*; PDB ID 6CFW) via their respective proton-pumping subunit Nqo14, MbsH, and MbhH. The alignment detail is shown in Fig. S4. The complexes are shown separately for clarity. All complexes have a similar peripheral arm (dashed outline) anchored either to the left end (complex I) or to the right end (MBS and MBH) of their respective membrane arm. Complex I has 3, MBS 2, and MBH 1 H^+^-pumping subunits. MBS and MBH, but not complex I, have a Na^+^ translocation module. **b** Superimposition of the two antiporter-like subunits of MBS (MbsH, MbsH’) and complex I (Nqo14, Nqo13), by aligning MbsH with Nqo14 as in (**a**). Top, side view with complex I Nqo12 TMH1-14 hidden; bottom, top view with complex I Nqo12 TMH1-14 shown as cartoon and marked by the transparent surface. Note that the length of helix HL is evolved to match the number of antiporter-like subunits present in MBS and complex I. **c** The potential Na^+^ and H^+^ translocation paths in MBS. The Na^+^ path and the adjacent H^+^ path together form the potential Na^+^/H^+^ antiporter module in the distal end of the membrane arm. The question mark above the proton path in the antiporter module indicates the tentative nature of the assignment. In the three H^+^ paths, key residues conserved with complex I are shown as sticks. In the Na^+^ path, key residues conserved with MBH and Mrp are shown as sticks. A series of charged and protonatable residues extend from MbsM to MbsH and MbsH’ to form the charged axis across the membrane interior.

In the proximal membrane module, the two largest subunits MbsH and MbsH’ are of similar fold, consistent with the idea from sequence comparison that MbsH’ is a gene-duplication product of MbsH^19^ (Supplementary Fig. 3b) that occurred prior to the evolution of MBH and MBS and, as discussed below, during the evolution of the Na^+^/H^+^ Mrp antiporter. MbsH and MbsH’ are homologous to the antiporter-like subunits of complex I (*T. thermophilus* Nqo12, 13 and 14) proposed for H^+^ pumping^1–3,5^. MbsH and MbsH’ each contain two five-TMH repeating units: TMHs 4-8 and TMHs 9-13 that are superimposable when one repeat is flipped upside down in the membrane (Supplementary Fig. 3b). Each 5-TMH unit features a discontinuous α-helix—TMH7 in the first repeat and TMH12 in the second repeat with conserved charged residues around the helical kink: E219 and K393 in MbsH, and K217 and K399 in MbsH’. Notably, the arrangement between MbsH and MbsH’ as a unit is strikingly similar to Nqo13 and Nqo14 of *T. thermophilus* complex I (Fig. 3a, b). As a result, a central charged axis across MbsH and MbsH’ (MbsH E138, K250, and H330; MbsH’ E128, K248, and H336) is formed that is conserved in complex I (Fig. 3c). These features are characteristic of the proton pumps that are found in mammalian and bacterial forms of complex I, which have been supported by structural studies, mutagenesis analysis, and recent molecular dynamics simulations^1–3,28–30^. We therefore propose that MbsH and MbsH’ each contains a H^+^ pump in MBS.

Interestingly, MbsH’ contains two extra TMHs (TMH15 and TMH16) that are connected by a long intervening loop (Loop_TMH15-16_) and the horizontal helix HL. The helix HL and its following TMH16 holds MbsH and MbsH’ together. This element is conserved across the different respiratory complexes, with the helix HL either shortened in MBH (with one antiporter-like subunit MbhH) or elongated in complex I (with three antiporter-like subunits) (Fig. 3a, b, Supplementary Fig. 4a), strongly supporting the modular evolution of these respiratory machineries^31^. Notably, Loop_TMH15-16_ and TMH15 of the C-terminal end of MbsH’ tie together the membrane-anchored peripheral module and the proton-pumping module (Supplementary Fig. 4a). This organization mode is conserved in the MBH complex, though MbsM and MbsH’ form a tighter interacting interface than the lipid-filled wide cleft between MbhM and MbhH in the MBH complex (Supplementary Fig. 4b)^6^. In contrast, in complex I, the membrane-anchored peripheral module is located on the opposite side of the membrane arm (Fig. 3a). However, in spite of this different architecture, a charged axis within the membrane-anchored peripheral arm (MbsM E203, E211, D196, E193, and E104) extends continuously into the proton-pumping module (MbsH and MbsH’) (Fig. 3c), similar to that proposed in complex I for energy transduction^1–3,32^.

### The tentative Na^+^/H^+^ antiporter module

Except for MbsH and MbsH’, the remainder of the proximal membrane module of MBS is composed of two layers of helix bundles next to MbsH: one layer composed of three TMHs of MbsD and the other layer composed of five TMHs—TMH1-2 of MbsE separated by TMH1-3 of MbsG in the middle (Supplementary Fig. 3a). This sub-region together with MbsH is conserved among MBH and complex I including key elements: charged residues E58 in MbsD, E37 in MbsG, and a π-bulge distortion in TMH3 of MbsD (Supplementary Fig. 4a and c). These are key features proposed for proton transfer that are conserved in Nqo10 and 11 in complex I and also in MbhD, G and E in MBH^2,3,6,33^, although the precise mechanism is still under debate^1,2,33^. This suggests a putative proton translocation path, termed Pc path for composite proton translocator. We note that the assignment of the Pc proton path is based on structural conservation among MBH, MBS, and complex I, and therefore, this path is tentative. The translocation stoichiometry of the H^+^ and Na^+^ is currently unknown and has yet to be experimentally determined with either Mrp, MBH, or MBS. The stoichiometry will help to determine if Pc is a functional proton path.

In the distal membrane module of MBS, MbsA at the far end contains two TMHs and a following ferredoxin-like domain and binds to the three 3-TMH subunits MbsB, C, and TMH3-6 of MbsE, which in turn are attached to MbsD of the proximal membrane module (Supplementary Fig. 3a). Notably, MbsB, C, and E contain three conserved regions that are rich in charged/polar residues and run across the interior of the membrane arm (Supplementary Fig. 4e). In addition, the conserved MbsC P83 introduces a break in the middle of the membrane of MbsC TMH3. These features are conserved in the Na^+^-translocation path in MBH and in Mrp, but not in complex I as complex I does not translocate Na^+^ ions (Fig. 3a, Supplementary Fig. 4d)^6,34^. This suggests a Na^+^-translocation path within this sub-region. This together with the adjacent H^+^ path (Pc) likely forms the Na^+^/H^+^ antiporter module in the distal end of the membrane arm (Fig. 3c).

**Fig. 4:**
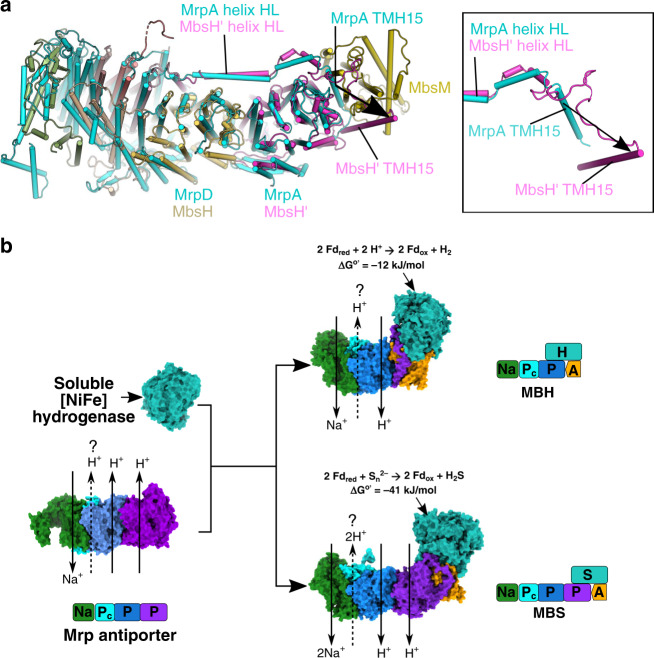
Evolutionary relationship of Mrp, MBH, and MBS. **a** Comparison of the MBS membrane arm with the Mrp structure (PDB ID: 6Z16). The structures are aligned by superimposing MbsH and MrpD. The MBS subunits are colored as Fig. 1c and the Mrp is in cyan. Mrp structure is similar to MBS, with the conserved helix HL holding the two antiporter-like subunits in both complexes (MrpA and MrpD; MbsH and MbsH’). Notably, the MbsH’ TMH15 immediately preceding helix HL moves a large distance, as compared to MrpA TMH15, to pack tightly against MbsM in order to accommodate MbsM in the MBS structure (see inset panel to the right). **b** A possible evolutionary pathway from Mrp to MBH and to MBS. Mrp antiporter: PDB ID 6Z16; cytoplasmic [NiFe] hydrogenase: PDB ID 2FRV; MBH: PDB ID 6CFW. The question mark above the proton path in each antiporter module indicates the tentative nature of the assignment. Shown above the MBH and MBS structures are the reductive reactions that they catalyze and the associated free energy changes. Located around each complex is the respective modular architectures of the Mrp-derived respiratory complexes. These modules are: Na (green), sodium ion path; P (blue and purple), H^+^ path(s) formed within a single subunit; Pc (cyan), composite H^+^ path formed by multiple small subunits; A (orange), membrane anchor for the peripheral arm; H (turquoise), peripheral arm for proton reduction; S (turquoise), peripheral arm for polysulfide reduction.

During the review of this manuscript, the structure of Mrp antiporter was published^35^. The structure enables a comparison between MBS and Mrp to obtain direct evolutionary insights between them (Fig. 4a). All subunits of the Mrp Na^+^/H^+^ antiporter have homologous counterparts in MBS (Supplementary Table 2). Consequently, the MBS membrane arm (excluding MbsM, which anchors the peripheral arm) shares a conserved architecture with Mrp (Fig. 4a). It is clear that the Mrp structure lacks the membrane-anchored peripheral arm and supports the modular evolution of respiratory complexes^31^. The emergence of MBS and MBH was likely a result of the combination of the Mrp antiporter and a peripheral module related to a cytoplasmic [NiFe] hydrogenase. MBH exhibits loss of the MrpA homolog resulting in fewer proton pumps while its peripheral arm retains the proton reduction activity. In contrast, MBS keeps all the Mrp homologs while its peripheral arm shows concomitant changes in the peripheral module to enable catalysis of the reduction of polysulfide rather than protons (Fig. 4b). Conceivably, both MBS and MBH are much more efficient in energy conservation as the redox reaction is now physically coupled with the ion translocation across the membrane.

### A three-loop system bridges the electron transfer site and the ion-pumping membrane arm

**Fig. 5:**
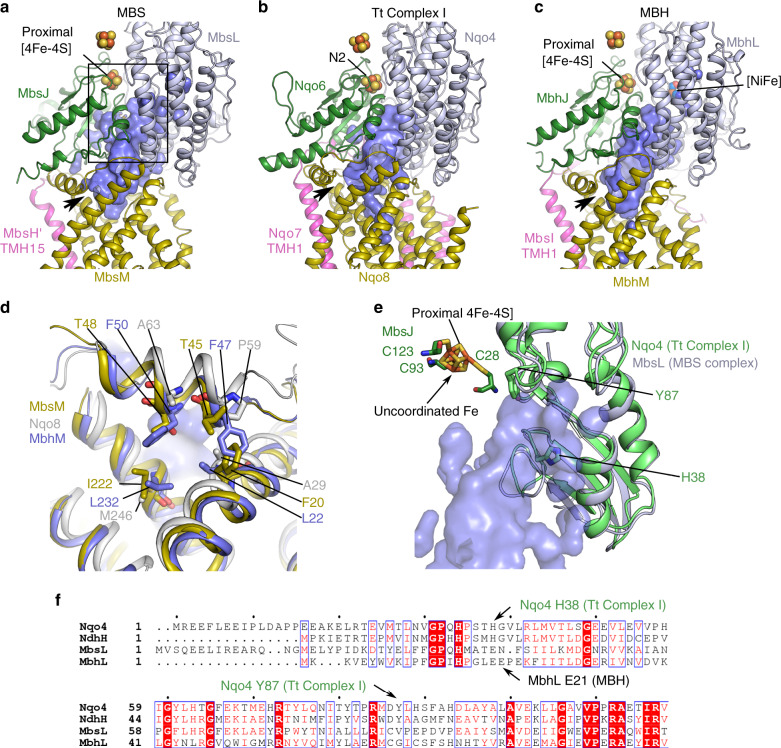
A comparison of the chamber at the active sites of MBS, MBH and complex I. The structures were overlaid based on MbsM, Nqo8, and MbhM and are shown individually. The chamber is similarly formed at the interface between the peripheral arm and its membrane anchor. The black arrow indicates the opening of the chamber proposed as the entrance of quinone in complex I studies. **a** MBS. **b** Complex I (PDB ID 4HEA). **c** MBH (PDB ID 6CFW). **d** A comparison of the chamber entry between MBS, MBH, and complex I indicate a wider opening in MBS and complex I than that in MBH. **e** Zoomed view of the end of the active site chamber, outlined as the boxed region in (**a**). The aligned Nqo4 of Tt complex I is shown as a green cartoon where two residues (H38 and Y87) proposed for the binding of the quinone headgroup are shown as sticks. **f** Sequence alignment of MbsL with its counterparts in different respiratory complexes (Nqo4 of Tt complex I, NdhH of NDH complex, MbhL of MBH complex I). Residues equivalent to Nqo4 H38 and Y87, proposed for quinone headgroup binding and protonation in complex I, and MbhL E21, the key residue for proton reduction in MBH complex, are all absent in MBS.

**Fig. 6:**
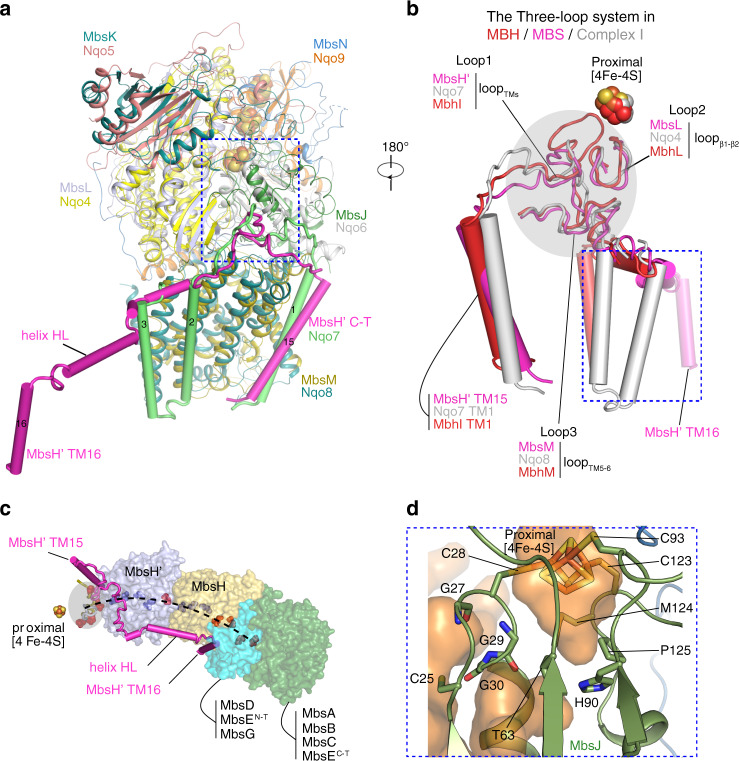
Membrane-anchored peripheral module of MBS. **a** Superimposition of the membrane-anchored peripheral modules of MBS and complex I by aligning MbsL with Nqo4. The aligned regions are shown in cartoons and colored as the text labels. Structural elements that bridge the peripheral module to the H^+^-pumping module (complex I Nqo7 and MBS MbsH’ TMH15-16, more details in Supplementary Fig. 4a) are shown as cylinders. **b** Between the peripheral arm and membrane arm are three interfacial loops, highlighted in gray shade, that are remarkably similar in MBH (red), MBS (magenta) and complex I (gray). The three loops are Loop_TMs_ (Loop 1), Loop_β1-β2_ (Loop 2), and Loop_TM5-6_ (Loop 3) and are adjacent to the proximal [4Fe-4S] cluster. The C-terminal TMs following Loop 1 (boxed in the dashed blue square) are least similar among the three-loop systems of MBH, MBS, and complex I. **c** The three-loop system (gray shade) is linked to the membrane ion translocation module in the semi-transparent surface through the C-terminal TMs following Loop 1. The dashed curve marks the central charge axis, a chain of red (negatively charged residues) and blue spheres (positively charged residues) spanning across the membrane arms. **d** Zoomed view of the area boxed with dashed blue square in (**a**) showing the coordination of the proximal [4Fe-4S]. MbsJ residues C28, C93 and C123 coordinate three irons of proximal [4Fe-4S] cluster, but MbsJ C25 is too far to coordinate the remaining iron. The semi-transparent orange surfaces show potential solvent-accessible chambers around the proximal cluster.

The architecture of the MBS cytoplasmic module closely resembles those of MBH and complex I (Supplementary Fig. 5). In MBS, a large hydrophobic chamber, similar in size to those in MBH and complex I, is formed by cytoplasmic MbsL, MbsJ, and their membrane anchor MbsM (Figs. 5a–c, 6a). The openings of the MBS and complex I hydrophobic chamber are wider than that of MBH allowing entry of hydrophobic substrates (polysulfide and quinone; Fig. 5d). The end of this chamber in MBS coincides with the binding site of the quinone headgroup defined by Nqo4 H38 and Y87 in complex I (Fig. 5e)^1^. However, these two residues for quinone headgroup binding and also for protonation in complex I are absent in MBS (Fig. 5f). Moreover, MbhL E21, the key residue for proton reduction in the MBH complex, is also absent in MBS^6^. These suggest a unique catalytic mechanism for MBS despite similar modular overall architecture, as we will cover in the next section.

This hydrophobic chamber containing the catalytic site of MBS in the peripheral arm is defined by three loops originating from MbsH’ (loop_TMH15-16_, now termed Loop 1), MbsL (loop_β1–β2_, Loop 2), and MbsM (loop_TMH5-6_, Loop 3) (Fig. 6b). The three-loop cluster connects the electron-transferring peripheral arm to the ion-pumping membrane arm and is strikingly similar in the three complexes (Fig. 6b)^1–3,6^. In MBS, Loop 1 via TM15 and TM16 of MbsH’ connects to its horizontal helix HL that spans two proton pumps (MbsH and MbsH’) (Supplementary Fig. 4a). Similarly in MBH, Loop 1 connects TM1 and TM2 of MbhI to its shorter helix HL that spans a single proton pump (MbhH). In complex I, Loop 1 and its preceding TM1 of Nqo7 locate the conserved membrane-anchored peripheral module to a different site of the membrane arm via TM2-3 of Nqo7 while the much longer helix HL of Nqo15 that spans three proton pumps (Nqo12, 13, and 14) is similar to MBS and MBH (Supplementary Fig. 4a). A central charged axis in MBS extending from the three loops across the membrane arm in parallel to helix HL is also found in both complex I and MBH (Figs. 3c, 6c)^1–3,6,32^. Clearly, the structural elements in MBH and complex I necessary for energy conservation are maintained in MBS. Therefore, we suggest that Loop1 and its preceding TM are a key structural feature that has been conserved across evolution in order to sustain the fundamental function of coupling the redox reaction in the peripheral arm with ion translocation in the membrane arm.

### The peripheral arm of MBS contains a uniquely coordinated [4Fe-4S] cluster

In MBS, the three [4Fe-4S] clusters form an efficient electron transfer pathway to the chamber from Fd (Fig. 1c inset**)**^36^. MbsN coordinates the distal and the medial [4Fe-4S] clusters (relative to the catalytic chamber; Supplementary Fig. 6a). The proximal (p-)[4Fe-4S] cluster in MBS and complex I is at the top of the hydrophobic chamber (Supplementary Fig. 6b) but, in stark contrast to that in complex I (and MBH), the MBS p-[4Fe-4S] is coordinated by only three rather than the expected four cysteines (C28, C93 and C123 of MbsJ; Fig. 6d).

**Fig. 7:**
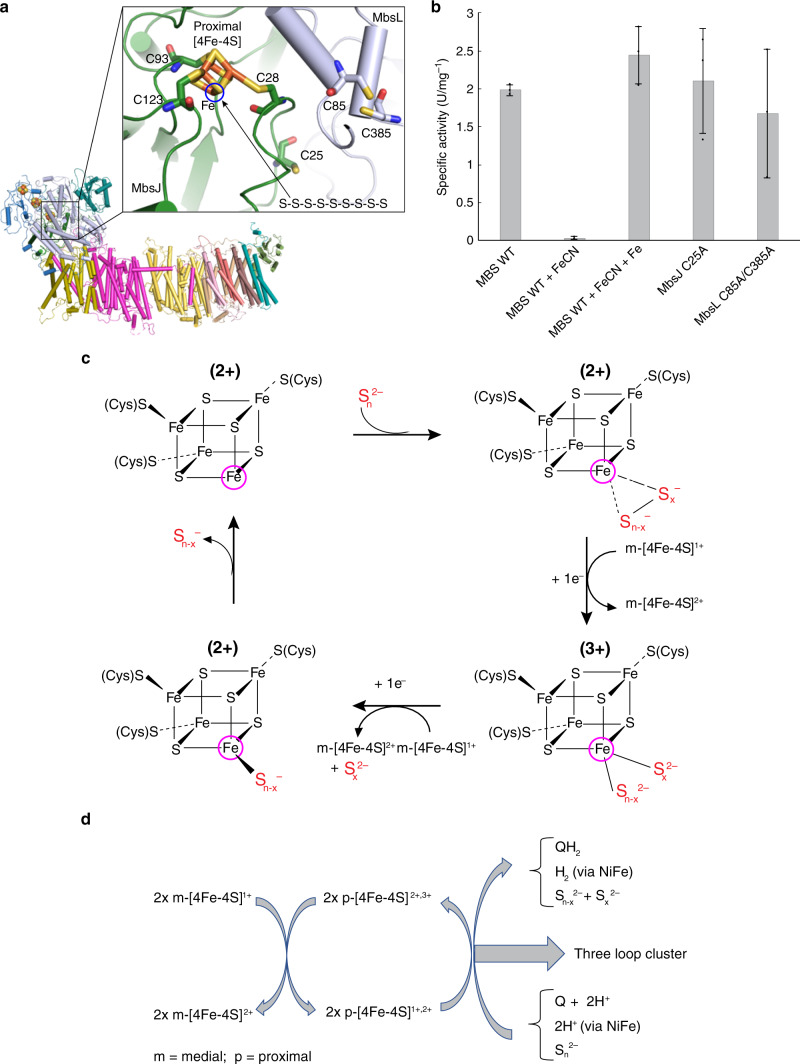
MBS catalyzed polysulfide reduction mechanism. **a** Cysteine residues within electron tunneling distance of the proximal [4Fe-4S] cluster. **b** Polysulfide reduction activity assay of purified WT and mutant C-MBS subcomplexes, demonstrating that MbsJ C25 and MbsL C85 and C385 are not involved in catalysis. Error bars are centered from the average value and represent standard deviation obtained using technical triplicate samples. **c** Proposed catalytic mechanism of polysulfide reduction for the MBS complex. **d** A sketch summarizing energy transduction in complex I, MBH, and MBS.

The different functions of the p-[4Fe-4S] clusters in these enzymes (Fig. 5b) are determined by a conserved structural motif containing a short N-terminal α-helix, a β-strand, a variable loop with two cysteines and another short α-helix (in MbsJ for MBS complex) (Supplementary Fig. 6b, c). In MBH and cytosolic [NiFe] hydrogenases^6,37^, the two cysteines are two residues apart and ideally positioned to both coordinate the proximal cluster, which donates electrons to the NiFe-catalytic site. In complex I, the two cysteines (C45 and C46 in Nqo6) are adjacent to each other enabling sequential dissociation upon proximal cluster reduction, which has been proposed to facilitate electron and proton transfer to the quinone although this is under debate^16,38,39^. In MBS, this structural motif is three residues shorter than it is in MBH, leaving one of the cysteines (MbsJ C25) no longer able to participate in cluster coordination (Fig. 6d, Supplementary Fig. 6c). Hence, one iron atom of the p-4Fe-4S] cluster in MBS has non-cysteinyl coordination (Supplementary Fig. 2j**)** and therefore catalytic potential. Besides the three [4Fe-4S] clusters, MBH has an additional proton-reducing [NiFe]-site, which is coordinated by four cysteines of MbhL (Supplementary Fig. 6d)^6,40^. Of these four cysteines, two are not present in MBS, leaving the two conserved residues (C85 and C385 in MbsL) adjacent to the proximal cluster (Fig. 7a). Complex I has lost all four, supporting the stepwise evolution of MBS and complex I by sequential loss of the four cysteines coordinating the [NiFe] center in MBH^31,41^.

In MBS, two potential polysulfide-reducing sites are close (~12 Å) to the p-[4Fe-4S] cluster: MbsJ C25 and MbsL C85/C385 (Fig. 7a). However, they are not involved in catalysis as the C25A MbsJ and the C85A/C385A MbsL mutant enzymes had comparable catalytic activities using the model substrate dimethyltrisulfide (DMTS) to that of the wild-type (WT) enzyme (1.8 ± 0.4 units/mg) (Fig. 7b). Cluster interconversion was used to provide evidence that the p-[4Fe-4S] cluster of MBS is itself reducing polysulfide, and doing so with a specific role for its unique non-cysteinyl coordinated Fe atom. The WT-enzyme was treated with potassium ferricyanide, a well-established procedure to remove non-cysteinyl coordinated Fe atom from an [4Fe-4S] cluster to generate a [3Fe-4S] cluster^21^. This caused an ~98% loss of activity (Fig. 7b). Subsequently incubating the enzyme under reducing conditions with ferrous iron, conditions that are known to restore the [4Fe-4S] cluster from a [3Fe-4S] cluster, the MBS activity was restored to its original value (Fig. 7b).

Further evidence for the proposed catalytic p-[4Fe-4S] cluster came from EPR spectroscopy. MBS from S^0^-grown cells gave rise to an intense free-radical EPR signal (*g* = 2.00) unaffected by chemical reduction (Supplementary Fig. 7a) but this was a purification artifact because the enzyme from non-S^0^ grown cells (in which transcription of the genes encoding MBS was controlled by a non-regulated promoter) did not exhibit this EPR signal (Supplementary Fig. 7b). Chemical reduction of MBS did lead to the appearance of a broad EPR signal (*g* ~ 1.94), consistent with three interacting and reduced [4Fe-4S] clusters (Supplementary Fig. 7b). After treatment with excess ferricyanide, the inactive enzyme (Fig. 7b) exhibited a complex EPR signal indicative of an oxidized *S* = 1/2 [3Fe-4S]^+^ cluster (*g* = 2.09, Supplementary Fig. 7c)^21^, supporting the conclusion that the p-[4Fe-4S] cluster is catalytic and that activity is reversibly lost upon removal of its unique iron. Importantly, chemically reduced *P. furiosus* MBH gave rise to an EPR signal (Supplementary Fig. 7d) similar to that of reduced MBS (Supplementary Fig. 7b), but when treated with ferricyanide under the same conditions used for MBS, no activity was lost and the MBH sample was EPR silent, consistent with the presence of only oxidized [4Fe-4S] clusters (Supplementary Fig. 7e). Unlike MBS, the p-[4Fe-4S] cluster of MBH is only involved in electron transfer and has full cysteinyl coordination, hence it is not susceptible to inactivation due to iron loss and oxidative cluster conversion.

### The proximal [4Fe-4S] cluster uses a novel, ancestral mechanism for sulfur reduction

The reduction of polysulfide by the p-[4Fe-4S] cluster in the hydrophobic pocket of MBS (Fig. 7a) is the first example in a biological system of reduction of an inorganic disulfide bond without the release of H_2_S (Eqs. 2 and 3, c.f. Eq. 5). Three types of enzymes use iron–sulfur clusters to indirectly facilitate disulfide bond cleavage; heterodisulfide reductase^42^, hydrogenase processing protein HydD^43^, and ferredoxin thioredoxin reductase (FTR)^44^. Based in part on FTR, we propose a new mechanism for the reduction of an internal disulfide (which could be either inorganic or organic) by MBS (Fig. 7c). The unique Fe atom of its p-[4Fe-4S]^2+^ cluster binds polysulfide (S_n_^2−^), accepts an electron from the medial (m-)[4Fe-4S]^1+^ cluster (Fig. 1c) and catalyzes the two-electron reduction of an internal disulfide, generating two smaller polysulfides (S_n-x_^2-^ and S_x_^2-^) and the superoxidized form of p-[4Fe-4S]^3+^. As in FTR, the reduction of the p-[4Fe-4S]^2+^ cluster actually leads to its superoxidation because two electrons are used to reduce the disulfide. Moreover, the cluster must be in the 3+ (rather than 2+) state to bind the two product polysulfides at the unique Fe site (Fig. 7c). The catalytic cycle is completed by reduction of p-[4Fe-4S]^3+^ by a second electron from m-[4Fe-4S]^1+^ (after its reduction by the distal [4Fe-4S]^1+^ cluster) and release of the two smaller polysulfides. This is the simplest mechanism yet proposed for the reduction of an S–S bond in either an inorganic or organic sulfide and is the first not requiring either protons or amino acid residues^42–44^. This reaction is clearly of fundamental significance in iron and sulfur-rich hydrothermal vent environments of the early earth and possibly in the origin of early life^21^, as well as in the subsequent evolution and expansion of iron–sulfur cluster functionality in the diverse array of modern-day iron–sulfur proteins^22,45^.

That catalysis by MBS does not require water or protons (Fig. 7c) is consistent with a highly hydrophobic pocket and the absence in MbsL of the well-defined proton transfer pathway to the NiFe site found in MbhL and other hydrogenases^6^. The requirement for binding two polysulfides (Fig. 7b) is superoxidation of the p-[4Fe-4S] cluster (3+), which is facilitated by the hydrophobic site, analogous to superoxidation of hydrophobic [4Fe-4S] clusters in high potential iron–sulfur proteins (HiPIPs)^46^. The spontaneous breakdown of tri- and disulfides to release H_2_S does require protons (Eq. 4) and must occur in the cytoplasm rather than the hydrophobic catalytic site (Fig. 7c). The di- and trisulfides produced by MBS do not generate H_2_S (or HS^−^), are stable in the hydrophobic pocket (where there are no protons or water), and H_2_S is only generated when they are released from the enzyme (Fig. 7c).

## Discussion

The standard state ΔG (pH 7.0) of the electron transfer reactions for MBS (41 kJ/mol/2e^−^) is more than twice that available to MBH (12 kJ/mol/2e^−^), and half of that available to complex I (81 kJ/mol/2e^−^)^9^, consistent with the pumping of two, one and four protons, respectively^1,2,7,8,47^. The MBS structure confirms that the transduction of redox energy into the spatially separated translocation of ions involves the three-loop cluster^1–3,31,48^ and a central charged axis^1–3,7,32^ (Fig. 6b, c), all highly conserved across evolution. The proposed MBS mechanism also shows that there are key fundamental differences in spite of a common overall means of energy transduction. In MBS, oxidation of the reduced m-[4Fe-4S]^1+^ cluster leads to superoxidation of the oxidized p-[4Fe-4S]^2+^ cluster (2^+^/3^+^ redox couple) but in MBH and complex I to reduction of the oxidized p-[4Fe-4S]^1+^ cluster (2^+^/1^+^ redox couple; Fig. 7c). Hence the loss of the NiFe site of an ancestral MBH through evolution generated a highly hydrophobic cavity enabling both MBS and complex I to use the p-[4Fe-4S] cluster to directly interact with and reduce hydrophobic substrates, albeit using different charge states of the cluster (Fig. 7d).

It is not clear how a change in the redox status of the m-[4Fe-4S] cluster is ultimately coupled to a change in the dynamics of the three-loop cluster, nor how the different amounts of free energy released (12–81 kJ/mol/2e^−^) lead to corresponding differences in the proton motive force that is generated (1–4 protons). It is interesting to speculate that MBS might conserve more (or less energy) if one proton pump (MbsH’ or equivalent, see Fig. 3c) were added (or inactivated, by mutagenesis) and that the cell yield of *P. furiosus* growing on S^0^ would be correspondingly higher (or lower). Such experiments are in progress. On a more fundamental level, the question yet to be answered is, as illustrated in Fig. 7d, how does m-[4Fe-4S]^1+^ oxidation coupled to a change in the conformation of the three-loop cluster lead to the conservation of different quantities of energy in these three types of the respiratory complex? In any event, the simple aprotic and abiotic catalytic mechanism of S–S bond reduction by MBS might be a modern remnant of an iron–sulfur world^21^ as well as the precursor to more complex mechanisms involving iron–sulfur clusters^42–44^ and those now found in the aerobic world, such as the reduction of quinones by complex I.

## Methods

### Purification of MBS

The MBS holoenzyme (S-MBS) was solubilized and purified anaerobically from *Pyrococcus furiosus* strain MW0491, in which a His_9_-tag had been engineered at the N terminus of the MbhJ subunit^19^. Frozen cells were lysed in 25 mM sodium phosphate, pH 7.5, containing 1 mM DTT and 50 μg/ml DNase I (5 mL per gram of frozen cells). After stirring for one hour, the cell-free extract was centrifuged at 100,000 × g for one hour. The supernatant was removed and the membranes were washed twice using 50 mM EPPS buffer, pH 8.0, containing 5 mM MgCl_2_, 50 mM NaCl, 10% (v/v) glycerol, 1 mM DTT, and 0.1 mM PMSF. The membrane pellet was collected by ultracentrifugation at 100,000 × g for one hour after each wash step. The washed membranes were resuspended in 50 mM Tris-HCl, pH 8.0, containing 5 mM MgCl_2_, 50 mM NaCl, 5% (v/v) glycerol, 1 mM DTT, and 0.1 mM PMSF. MBS was solubilized by adding n-dodecyl-β-D-maltoside (DDM, Inalco) to 3% (w/v) followed by incubation at 4 °C for 16 hours. The solubilized membranes were centrifuged at 100,000 × *g* for 1 h. The supernatant was applied to a 5 mL His-Trap crude FF Ni-NTA column (GE Healthcare) while diluting it 10-fold with buffer A (25 mM sodium phosphate, 300 mM NaCl, pH 7.5, containing 1 mM DTT and 0.03% DDM). The column was washed with 10 column volumes of buffer A and the bound protein was eluted with a 20-column volume gradient from 0 to 100% buffer B (buffer A containing 500 mM imidazole). The eluted protein was further purified by applying it to a 1-mL His-Trap HP Ni-NTA column (GE Healthcare) while diluting it 5-fold with buffer A. A 30-column volume gradient from 0 to 100% buffer B was used to elute the bound protein. The MBS sample was concentrated and further purified using a Superose 6 10/300 GL column (GE Healthcare) equilibrated with 50 mM Tris-HCl, pH 8.2, containing 300 mM NaCl, 2 mM sodium dithionite, and 0.03% DDM.

### *P. furiosus* strain construction

The strains used in this study are summarized in Supplementary Table 3. The genetically tractable *P. furiosus* strain COM1 was used for the genetic manipulation of MBS^49^. An insertion cassette was amplified using overlapping PCR^50^. The upstream flanking region (UFR), which also contained the selection marker (P_gdh_-*pyrF*) and the promoter of the gene encoding the S-layer protein (P_slp_), and the downstream flanking region (DFR), were amplified from MW0567 gDNA. Mutagenesis was carried out using site-directed mutagenesis, with primers designed to amplify the MbsJ^C25A^ mutation. The primers used are listed in Supplementary Table 4. The fragments were assembled using overlapping PCR and the insertion cassette was transformed into the intergenic space between PF0265 and PF0266 of MW0011. The transformants were grown as previously described^49^ and the PCR-confirmed colonies were sequence verified using Sanger sequencing (Genewiz).

### Purification of C-MBS, activity assays, and EPR methods

The three C-MBS subcomplexes (WT, MbsL^C85A/C385A^, MbsJ^C25A^) were purified anaerobically as described previously^19^. Frozen cells were lysed in 25 mM sodium phosphate, pH 7.5, containing 1 mM DTT and 50 µg/µL DNase I (5 mL per gram of frozen cells). After stirring for one hour, the cell-free extract was centrifuged at 100,000 × g for one hour. The supernatant was applied directly to a 5 mL His-Trap FF Ni-NTA column (GE Healthcare) by diluting it 10-fold with 25 mM sodium phosphate, pH 7.5 containing 300 mM NaCl and 1 mM DTT (Buffer C). The column was equilibrated with Buffer C before loading the sample. The column was washed with 5 column volumes of Buffer C and the bound protein was eluted with a 20-column volume gradient from 0 to 100% Buffer D (Buffer C containing 500 mM imidazole).

All activity assays were carried out at 80 °C using anaerobic sealed cuvettes as described previously^19^. The dimethyl trisulfide (DMTS) reduction assay used a 2 mL reaction mixture containing 100 mM 3-(N-morpholino) propanesulfonic acid (MOPS), pH 7.5, and 150 mM NaCl. After pre-heating to 80 °C, 1 mM methyl viologen reduced by titanium citrate and 2 mM DMTS were added and the reaction was initiated by the addition of enzyme. For inactivation by cluster conversion, the enzyme (2 mg/mL in 25 mM sodium phosphate, pH 7.5) was incubated anaerobically with potassium ferricyanide (1 mM) for ~90 h at room temperature. The sample was then buffer exchanged to remove excess ferricyanide before measuring activity. To activate the enzyme, a mixture of sodium dithionite (1 mM) and FeCl_3_ (1 mM) were added and after ~90 h at room temperature, the sample was buffer exchanged to remove the excess reagent. Enzyme activity was measured by the reduction of DMTS by monitoring the oxidation of methyl viologen at 600 nm (ε = 8.25 mM^−1^ cm^−1^) as previously described^19^. One unit of activity is defined as 1 µmol of DMTS reduced per min.

All samples for spectroscopic studies were prepared under strictly anaerobic conditions. X-band (~9.6 GHz) EPR spectra were recorded using a Bruker EMXplus CW EPR spectrometer controlled with a Bruker PremiumX Ultra low noise microwave bridge, equipped with a cryogen-free ColdEdge stinger system and a Lakeshore temperature controller.

### Cryo-EM analysis and data acquisition

MBS was purified using detergent DDM and was concentrated to ~5 mg/ml. Cryo-EM grids were prepared under the aerobic conditions and the processes took 20–30 min (MBS in air has a half-life of 19 h^19^). In brief, three-microliter aliquots of the sample were applied to glow-discharged Quantfoil R 1.2/1.3 gold grids (300 mesh). The grids were blotted for 3 s at 10 °C with 100% humidity and were flash-frozen in liquid ethane using an FEI Vitrobot Mark IV device. Cryo-EM data were recorded on a K2 camera positioned post a GIF quantum energy filter in a 300 kV FEI Titan Krios electron microscopy. Two datasets were automatically collected with Serial EM 3.7beta and FEI EPU software package, respectively, containing 8352 and 6355 movies respectively. Micrographs were recorded in counting mode at a nominal magnification of 130,000× with a pixel size of 1.03 Å on sample. Defocus values varied from −1.1 to −3 μm. The dose rate was 10 electrons per pixel per second. A total exposure of 6 s was dose-fractionated into 30 sub-frames, resulting in a total accumulated dose of 52 electrons per Å^2^.

### Image processing

The two datasets collected on Titan Krios were processed using a similar strategy. Dose-fractionated movies with a physical pixel size of 1.03 Å were motion-corrected and dose-weighted with MotionCor2 1.1^51,52^. The contrast transfer function (CTF) parameter for individual micrograph was estimated by CTFFIND4.1.10^53^. Further processing steps were carried out using RELION-3.0^54^. For each dataset, a small set of particles were manually picked and were subjected to 2D classification. This generated templates for further reference-based automatic particle picking. Particle sorting and reference-free 2D classification were applied to the auto-picked particles for the removal of bad particles, resulting in 676,320 and 667,428 particles from the two respective datasets. The further 3D classification was performed on these cleaned-up particles using an ab initio map generated by cryoSPARC v2 as the initial model^55^. For each dataset, three of four 3D classes were combined to get 467,320 and 419,868 good particles, respectively. Those were subjected to another round of 3D classification. One 3D class from each dataset with the best density features were combined. The combined dataset (203,673 particles) were used for 3D auto-refinement following CTF refinement, resulting in a map with an overall resolution of 4.0 Å when twofold symmetry was applied. We note that most regions, particularly in the peripheral arm and the proximal membrane module, are of substantially better resolution in the range of 3.0–3.5 Å. Some strategies including applying C1 symmetry, masking out detergent micelle or expanding the symmetry failed to improve the map. We therefore reasoned that the low overall resolution (4 Å) may be due to the partially flexible distal membrane region. The resolution of the reconstructed map was estimated based on the gold-standard Fourier shell correlation 0.143 criterion^56^. The final map was corrected for the modulation transfer function of the detector and sharpened by applying a negative B-factor, estimated by the post-processing procedure in RELION-3.0. The estimate of Local resolution distribution was calculated using ResMap 1.1.5^57^.

### Model building and refinement

The initial models of the distal module (MbsA-C, MbsE TMH3-6) and membrane-anchored hydrogenase module (MbsJ-N) were generated with the SWISS-MODEL server^58^ using the structure of *P. furiosus* MBH complex (PDB ID 6CFW) as the template. The initial model of the proximal module (MbsD, MbsE TMH3-6, MbsG, MbsH, and MbsH’) were generated using the structure of *T. thermophilus* complex I as a template (PDB ID 4HEA). These models were individually rigid-body docked into the 3D density map using the Fit-in-map function in Chimera 1.8^59^. These fitted models were improved by manual adjustments and rebuilding in Coot 0.8.9^60^. The rebuilding process was aided by the good density features of α helices and many bulky residues such as Phe, Trp, Tyr, and Arg. The clusters within the peripheral arm were modeled based on their electron densities and also the homology of cluster-coordinating subunits with complex I and MBH. The real-space refinement of the MBS complex model against the cryo-EM map was performed using the phenix.real_space_refine in PHENIX 1.15^61^. MolProbity 4.1 (Duke University) was used to assess the final model^62^. Chimera and PyMOL 1.8 (Schrödinger, LLC.) were used to prepare the Figures. Statistics of the 3D reconstruction and model refinement were provided in Supplementary Table 1. Since all the subunits of Mrp antiporters have their counterparts in MBS (Supplementary Table 2), the structure of *B. subtilis* Mrp antiporter was modeled based on the MBS structure using the SWISS-MODEL server^58^.

### Reporting summary

Further information on research design is available in the Nature Research Reporting Summary linked to this article.

## Supplementary information

Supplementary Information

Reporting summary

## Data Availability

The accession number for the atomic coordinates reported in this paper is PDB ID 6U8Y. The accession number for the EM density map reported in this paper is EMD-20692. Source data are provided with this paper. Other data are available from the corresponding authors upon reasonable request. Source data are provided with this paper.

## Source data

Source data

## References

[1] Baradaran R, Berrisford JM, Minhas GS, Sazanov LA (2013). Crystal structure of the entire respiratory complex I. Nature.

[2] Zickermann V (2015). Structural biology. Mechanistic insight from the crystal structure of mitochondrial complex I. Science.

[3] Zhu J, Vinothkumar KR, Hirst J (2016). Structure of mammalian respiratory complex I. Nature.

[4] Fiedorczuk K (2016). Atomic structure of the entire mammalian mitochondrial complex I. Nature.

[5] Guo R, Zong S, Wu M, Gu J, Yang M (2017). Architecture of human mitochondrial respiratory megacomplex I2III2IV2. Cell.

[6] Yu H (2018). Structure of an ancient respiratory system. Cell.

[7] Sazanov LA (2015). A giant molecular proton pump: structure and mechanism of respiratory complex I. Nat. Rev. Mol. Cell Biol..

[8] Hirst J (2013). Mitochondrial complex I. Annu. Rev. Biochem..

[9] Schut GJ (2016). The role of geochemistry and energetics in the evolution of modern respiratory complexes from a proton-reducing ancestor. Biochim Biophys. Acta.

[10] Letts JA, Sazanov LA (2017). Clarifying the supercomplex: the higher-order organization of the mitochondrial electron transport chain. Nat. Struct. Mol. Biol..

[11] Agip, A. A., Blaza, J. N., Fedor, J. G. & Hirst, J. Mammalian respiratory Complex I through the lens of cryo-EM. *Ann. Rev. Biophys.* **48**, 165–184 (2019). 10.1146/annurev-biophys-052118-115704 30786232

[12] Sapra R, Bagramyan K, Adams MW (2003). A simple energy-conserving system: proton reduction coupled to proton translocation. Proc. Natl Acad. Sci. USA.

[13] Mayer F, Muller V (2014). Adaptations of anaerobic archaea to life under extreme energy limitation. FEMS Microbiol. Rev..

[14] Kim YJ (2010). Formate-driven growth coupled with H(2) production. Nature.

[15] Lim JK, Mayer F, Kang SG, Muller V (2014). Energy conservation by oxidation of formate to carbon dioxide and hydrogen via a sodium ion current in a hyperthermophilic archaeon. Proc. Natl Acad. Sci. USA.

[16] Laughlin TG, Bayne AN, Trempe JF, Savage DF, Davies KM (2019). Structure of the complex I-like molecule NDH of oxygenic photosynthesis. Nature.

[17] Nisbet EG, Sleep NH (2001). The habitat and nature of early life. Nature.

[18] McTernan PM (2014). Intact functional fourteen-subunit respiratory membrane-bound [NiFe]-hydrogenase complex of the hyperthermophilic archaeon *Pyrococcus furiosus*. J. Biol. Chem..

[19] Wu CH, Schut GJ, Poole FL, Haja DK, Adams MWW (2018). Characterization of membrane-bound sulfane reductase: a missing link in the evolution of modern day respiratory complexes. J. Biol. Chem..

[20] Schut GJ, Bridger SL, Adams MW (2007). Insights into the metabolism of elemental sulfur by the hyperthermophilic archaeon *Pyrococcus furiosus*: characterization of a coenzyme A- dependent NAD(P)H sulfur oxidoreductase. J. Bacteriol..

[21] Conover RC (1990). Spectroscopic characterization of the novel iron-sulfur cluster in *Pyrococcus furiosus* ferredoxin. J. Biol. Chem..

[22] Schicho RN, Ma K, Adams MW, Kelly RM (1993). Bioenergetics of sulfur reduction in the hyperthermophilic archaeon *Pyrococcus furiosus*. J. Bacteriol..

[23] Enemark JH, Cosper MM (2002). Molybdenum enzymes and sulfur metabolism. Met. Ions Biol. Syst..

[24] Jormakka M (2008). Molecular mechanism of energy conservation in polysulfide respiration. Nat. Struct. Mol. Biol..

[25] Efremov RG, Sazanov LA (1817). The coupling mechanism of respiratory complex I - a structural and evolutionary perspective. Biochim Biophys. Acta.

[26] Hedderich R (2004). Energy-converting [NiFe] hydrogenases from archaea and extremophiles: ancestors of complex I. J. Bioenerg. Biomembr..

[27] Schut GJ, Boyd ES, Peters JW, Adams MW (2013). The modular respiratory complexes involved in hydrogen and sulfur metabolism by heterotrophic hyperthermophilic archaea and their evolutionary implications. FEMS Microbiol Rev..

[28] Di Luca A, Gamiz-Hernandez AP, Kaila VRI (2017). Symmetry-related proton transfer pathways in respiratory complex I. Proc. Natl Acad. Sci. USA.

[29] Kaila, V. R. I. Long-range proton-coupled electron transfer in biological energy conversion: towards mechanistic understanding of respiratory complex I. *J. R. Soc., Interface* **15**, 20170916 (2018). 10.1098/rsif.2017.0916 PMC5938582 29643224

[30] Euro L, Belevich G, Verkhovsky MI, Wikstrom M, Verkhovskaya M (2008). Conserved lysine residues of the membrane subunit NuoM are involved in energy conversion by the proton-pumping NADH:ubiquinone oxidoreductase (Complex I). Biochim Biophys. Acta.

[31] Brandt U (2019). Adaptations of an ancient modular machine. Science.

[32] Agip AA (2018). Cryo-EM structures of complex I from mouse heart mitochondria in two biochemically defined states. Nat. Struct. Mol. Biol..

[33] Efremov RG, Sazanov LA (2011). Structure of the membrane domain of respiratory complex I. Nature.

[34] Morino M (2010). Single site mutations in the hetero-oligomeric Mrp antiporter from alkaliphilic Bacillus pseudofirmus OF4 that affect Na+/H+ antiport activity, sodium exclusion, individual Mrp protein levels, or Mrp complex formation. J. Biol. Chem..

[35] Steiner, J. & Sazanov, L. Structure and mechanism of the Mrp complex, an ancient cation/proton antiporter. *Elife* **9**, e59407 (2020). 10.7554/eLife.59407 PMC7419157 32735215

[36] Page CC, Moser CC, Chen X, Dutton PL (1999). Natural engineering principles of electron tunnelling in biological oxidation-reduction. Nature.

[37] Volbeda, A. et al. Structure of the [NiFe] hydrogenase active site: evidence for biologically uncommon Fe ligands. *J. Am. Chem. Soc.* **118**, 12989–12996 (1996).

[38] Schuller JM (2019). Structural adaptations of photosynthetic complex I enable ferredoxin-dependent electron transfer. Science.

[39] Berrisford JM, Sazanov LA (2009). Structural basis for the mechanism of respiratory complex I. J. Biol. Chem..

[40] Fontecilla-Camps JC, Volbeda A, Cavazza C, Nicolet Y (2007). Structure/function relationships of [NiFe]- and [FeFe]-hydrogenases. Chem. Rev..

[41] Kashani-Poor N, Zwicker K, Kerscher S, Brandt U (2001). A central functional role for the 49-kDa subunit within the catalytic core of mitochondrial complex I. J. Biol. Chem..

[42] Wagner T, Koch J, Ermler U, Shima S (2017). Methanogenic heterodisulfide reductase (HdrABC-MvhAGD) uses two noncubane [4Fe-4S] clusters for reduction. Science.

[43] Watanabe S (2007). Crystal structures of [NiFe] hydrogenase maturation proteins HypC, HypD, and HypE: Insights into cyanation reaction by thiol redox signaling. Mol. Cell.

[44] Walters EM (2009). Role of histidine-86 in the catalytic mechanism of ferredoxin: thioredoxin reductase. Biochemistry.

[45] Torres-Bacete J, Nakamaru-Ogiso E, Matsuno-Yagi A, Yagi T (2007). Characterization of the NuoM (ND4) subunit in *Escherichia coli* NDH-1: conserved charged residues essential for energy-coupled activities. J. Biol. Chem..

[46] Dey A (2007). Solvent tuning of electrochemical potentials in the active sites of HiPIP versus ferredoxin. Science.

[47] Galkin, A., Drose, S. & Brandt, U. The proton pumping stoichiometry of purified mitochondrial complex I reconstituted into proteoliposomes. *Biochim Biophys. Acta* **1757**, 1575–1581 (2006). 10.1016/j.bbabio.2006.10.001 17094937

[48] Cabrera-Orefice A (2018). Locking loop movement in the ubiquinone pocket of complex I disengages the proton pumps. Nat. Commun..

[49] Lipscomb GL (2011). Natural competence in the hyperthermophilic archaeon *Pyrococcus furiosus* facilitates genetic manipulation: construction of markerless deletions of genes encoding the two cytoplasmic hydrogenases. Appl Environ. Microbiol..

[50] Bryksin AV, Matsumura I (2010). Overlap extension PCR cloning: a simple and reliable way to create recombinant plasmids. Biotechniques.

[51] Grant T, Grigorieff N (2015). Measuring the optimal exposure for single particle cryo-EM using a 2.6 A reconstruction of rotavirus VP6. Elife.

[52] Zheng SQ (2017). MotionCor2: anisotropic correction of beam-induced motion for improved cryo-electron microscopy. Nat. Methods.

[53] Rohou A, Grigorieff N (2015). CTFFIND4: Fast and accurate defocus estimation from electron micrographs. J. Struct. Biol..

[54] Kimanius, D., Forsberg, B. O., Scheres, S. H. & Lindahl, E. Accelerated cryo-EM structure determination with parallelisation using GPUs in RELION-2. *Elife* **5**, e18722 (2016). 10.7554/eLife.18722 PMC5310839 27845625

[55] Punjani A, Rubinstein JL, Fleet DJ, Brubaker MA (2017). cryoSPARC: algorithms for rapid unsupervised cryo-EM structure determination. Nat. Methods.

[56] Rosenthal PB, Henderson R (2003). Optimal determination of particle orientation, absolute hand, and contrast loss in single-particle electron cryomicroscopy. J. Mol. Biol..

[57] Kucukelbir A, Sigworth FJ, Tagare HD (2014). Quantifying the local resolution of cryo-EM density maps. Nat. Methods.

[58] Arnold K, Bordoli L, Kopp J, Schwede T (2006). The SWISS-MODEL workspace: a web-based environment for protein structure homology modelling. Bioinformatics.

[59] Pettersen EF (2004). UCSF Chimera-a visualization system for exploratory research and analysis. J. Comput. Chem..

[60] Emsley P, Lohkamp B, Scott WG, Cowtan K (2010). Features and development of Coot. Acta Crystallogr. D. Biol. Crystallogr..

[61] Adams PD (2010). PHENIX: a comprehensive Python-based system for macromolecular structure solution. Acta Crystallogr. D. Biol. Crystallogr..

[62] Chen VB (2010). MolProbity: all-atom structure validation for macromolecular crystallography. Acta Crystallogr. D. Biol. Crystallogr..

