# Author Correction: Structure of the respiratory MBS complex reveals iron-sulfur cluster catalyzed sulfane sulfur reduction in ancient life

**DOI:** 10.1038/s41467-021-26141-x

**Published:** 2021-09-30

**Authors:** Hongjun Yu, Dominik K. Haja, Gerrit J. Schut, Chang-Hao Wu, Xing Meng, Gongpu Zhao, Huilin Li, Michael W. W. Adams

**Affiliations:** 1 Structural Biology Program, Van Andel Institute, Grand Rapids, MI USA; 2 Department of Biochemistry and Molecular Biology, University of Georgia, Athens, GA USA; 3 Present Address: Department of Biochemistry and Molecular Biology, School of Basic Medicine and Tongji Medical College, Huazhong University of Science and Technology, Wuhan, China

**Keywords:** Metalloproteins, Cryoelectron microscopy, Metalloproteins, Cryoelectron microscopy

Correction to: *Nature Communications* 10.1038/s41467-020-19697-7, published online 23 November 2020.

The original version of this Article omitted the following from the Acknowledgements: The EPR spectrometer was funded by an instrument award (CHE-1827968) from the National Science Foundation.

This has now been corrected in both the PDF and HTML versions of the Article.

