# Supplementary material for: Structure of the respiratory MBS complex reveals iron-sulfur cluster catalyzed sulfane sulfur reduction in ancient life
Source: Nat Commun. 2020 Nov 23;11:5953. doi: 10.1038/s41467-020-19697-7 (PMC7684303; doi:10.1038/s41467-020-19697-7)
Supplement: Supplementary file 1 — Supplementary Information [file 41467_2020_19697_MOESM1_ESM.pdf]

Supplementary Information

Structure of the respiratory MBS complex reveals iron-sulfur cluster catalyzed sulfane sulfur reduction in ancient life

By Yu et al.

This document contains:

Supplemental Tables 1 - 4
Supplemental Figures 1 – 7

Supplementary Table 1. Cryo-EM data collection and refinement statistics

	MBS complex
**Data collection**	
Microscope	FEI Titan Krios
Voltage (kV)	300
Detector	Gatan K2 Summit
Electron dose (e^-/Å^2)	52
Pixel size (Å)	1.03
Defocus range (–μm)	1.1 – 3.0
**Reconstruction**	
Software	RELION 3.0
Particles for final refinement	203,673
Final Resolution (Å)	4.0
Map-sharpening B factor (Å^2)	–187.49
**Model composition**	
Peptide chains	26
Residues	6140
[4Fe-4S]	6
**R.m.s. deviations**	
Bond lengths (Å)	0.008
Bond angles (°)	1.205
**Ramachandran plot**	
Favored (%)	87.6
Outlier (%)	0.3
**Validation**	
Molprobity score	2.1
Rotamer outlier (%)	1.9
Clashscore	4.7

Supplementary Table 2. Homologous counterparts among respiratory Complex I (*T. thermophilus*), *B. subtilis* Mrp H^{+}/Na^{+} antiporter, *P. furiosus* MBH, and *P. furiosus* MBS

Module proposed from MBH	*P. furiosus* MBH complex	*P. furiosus* Mbs complex	*B. subtilis* Mrp complex	*T. Thermophilus* Complex I	Module of Complex I
-	-	-	-	Nqo15/Nqo16	-
-	-	-	-	Nqo1/Nqo2/ Nqo3	N-module
Membrane-anchored hydrogenase module	MbhJ	MbsJ	-	Nqo6	Q-module
	MbhK	MbsK	-	Nqo5	
	MbhL	MbsL	-	Nqo4	
	MbhN	MbsN	-	Nqo9	
	MbhM	MbsM	-	Nqo8	P-module
Linker between hydrogenase module and ion-translocation modules	MbhI N-terminal	MbsH' TM15-16	MrpA TM15-16	Nqo7 N-terminal	
	MbhI C-terminal			Nqo12 C-terminal	
Proton translocation module	MbhD	MbsD	MrpA TM17-21	Nqo10	
	MbhE	MbsE TM1-2			
	MbhG	MbsG	MrpC	Nqo11	
	MbhH	MbsH	MrpD	Nqo14	
	-	MbsH' TM1-14	MrpA TM1-14	Nqo13	
	-	-	-	Nqo12	
Sodium translocation module	MbhF	MbsE TM3-6	MrpB	-	-
	MbhA	MbsA	MrpE	-	
	MbhB	MbsB	MrpF	-	
	MbhC	MbsC	MrpG	-	

Supplementary Table 3. Strains constructed and used in this study

Trivial Name	Strain Name	Description	Ref.
COM1	MW0002	Δ*pyrF*	52
MbsJ-His	MW0491	Δ*pyrF*::P_{gdh}*pyrF* P_{slp} 9x His *mbsJ*	5
ΔMbsL	MW0011	Δ*pyrF* Δ*mbsL*	21
$MbsL^{WT}$	MW0567	Δ*pyrF* Δ*mbsL*::P_{gdh}*pyrF* P_{slp} 9x His *mbsL-N*	5
$MbsL^{C85A/C385A}$	MW0572	Δ*pyrF* Δ*mbsL*::P_{gdh}*pyrF* P_{slp} 9x His *mbsL-N* ($MbsL^{C85A/C385A}$)	5
$MbsJ^{C25A}$	MW0584	Δ*pyrF* Δ*mbsL*::P_{gdh}*pyrF* P_{slp} 9x His *mbsL-N* ($MbsJ^{C25A}$)	This study

Supplementary Table 4: Primers used in this study

Lab Name	Target	Sequence (5’ → 3’)
CH142	C-MBS UFR F	TCGAGGAGTACAACATCTTTGCCCAGGC
CH151	C-MBS DFR R	GTTTGGGTTGTGAGAGAAAGCCAAAAGCTTTCTCC
DH51	MbsJ C25A F	AAGAGTCTTTGGATTGTTGCATTTGCTACCGGATGTGGTGGAATAGAGAT
DH52	MbsJ C25A R	ATCTCTATTCCACCACATCCGGTAGCAAATGCAACAATCCAAAGACTCTT

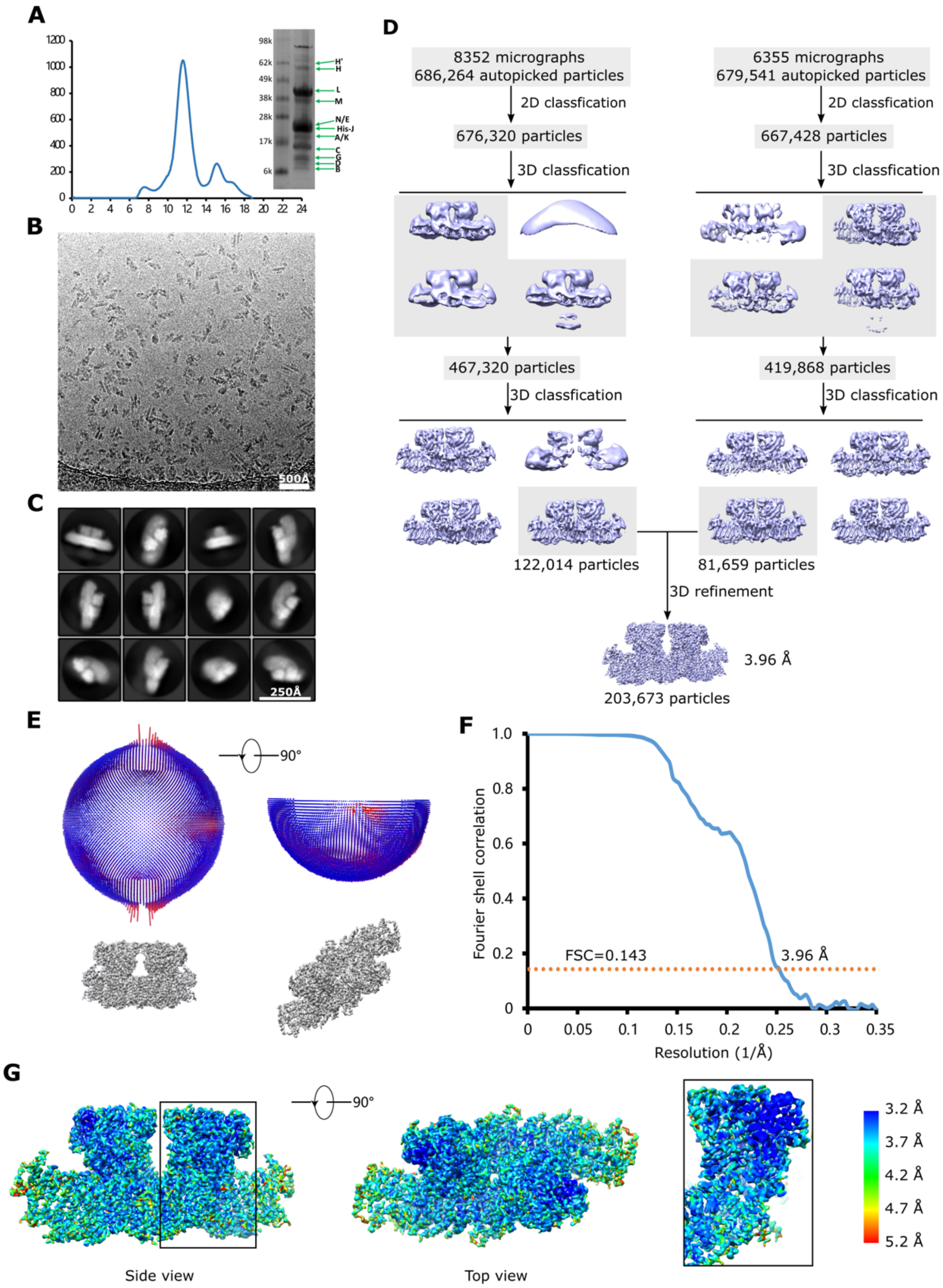

Supplementary Figure 1. Sample preparation and Cryo-EM of *P. furiosus* MBS. (A) A representative profile of gel filtration chromatography (Superose 6 10/300 GL column) of MBS purified with the detergent n-Dodecyl β-D-maltoside. The gel filtration were performed two times, for each sample used for data collection. Inset: SDS-PAGE of the purified MBS sample showing the presence of all thirteen MBS subunits as labeled to the right. Molecular weight markers in kDa are labeled to the left. (**B**) Representative cryo-EM micrograph of the MBS

sample. We collected two datasets, one with 8352 and the other with 6355 micrographs. (**C**) Representative 2D class averages. A total of 203,673 particles were selected after 2D and 3D classifications. (**D**) The workflow of cryo-EM data processing. (**E**) Two views of the angular distribution of all particles used in the final 3D reconstruction (top panel). Shown below is the corresponding view of the 3D map. (**F**) The gold-standard Fourier shell correlation curve of the 3D map. (**G**) Local resolution of the cryo-EM 3D map calculated with ResMap. Inset: cut-open view showing the internal resolution range of the boxed area of the side view in the left most panel.

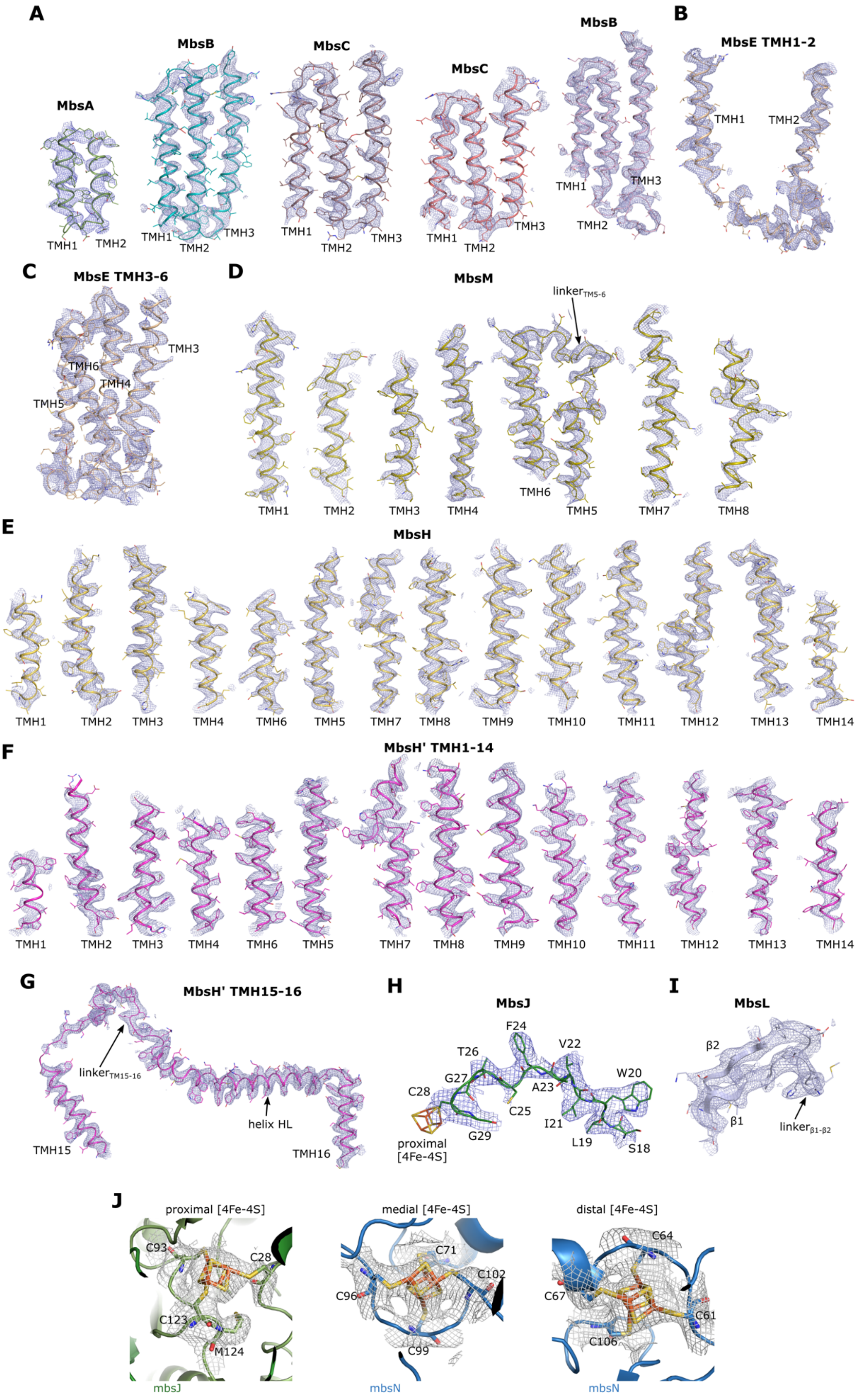

Supplementary Figure 2. A gallery of example regions of the EM density map and the

atomic model fitted in the map. These regions include all 58 TMHs of all membrane subunits (**A-G**), Loop$_{TMH5-6}$ of MbsM (**D**), Loop$_{TMH15-16}$, helix HL of MbsH' (**G**), the N-terminal part of MbsJ containing C25 that unexpectedly does not coordinate the proximal [4Fe-4S] cluster (**H**), and the N-terminal β-sheets of MbsL connected by Loop$_{\beta1-\beta2}$ (**I**). (**J**) The EM densities around the three [4Fe-4S] clusters are rendered in surface view at the same display threshold.

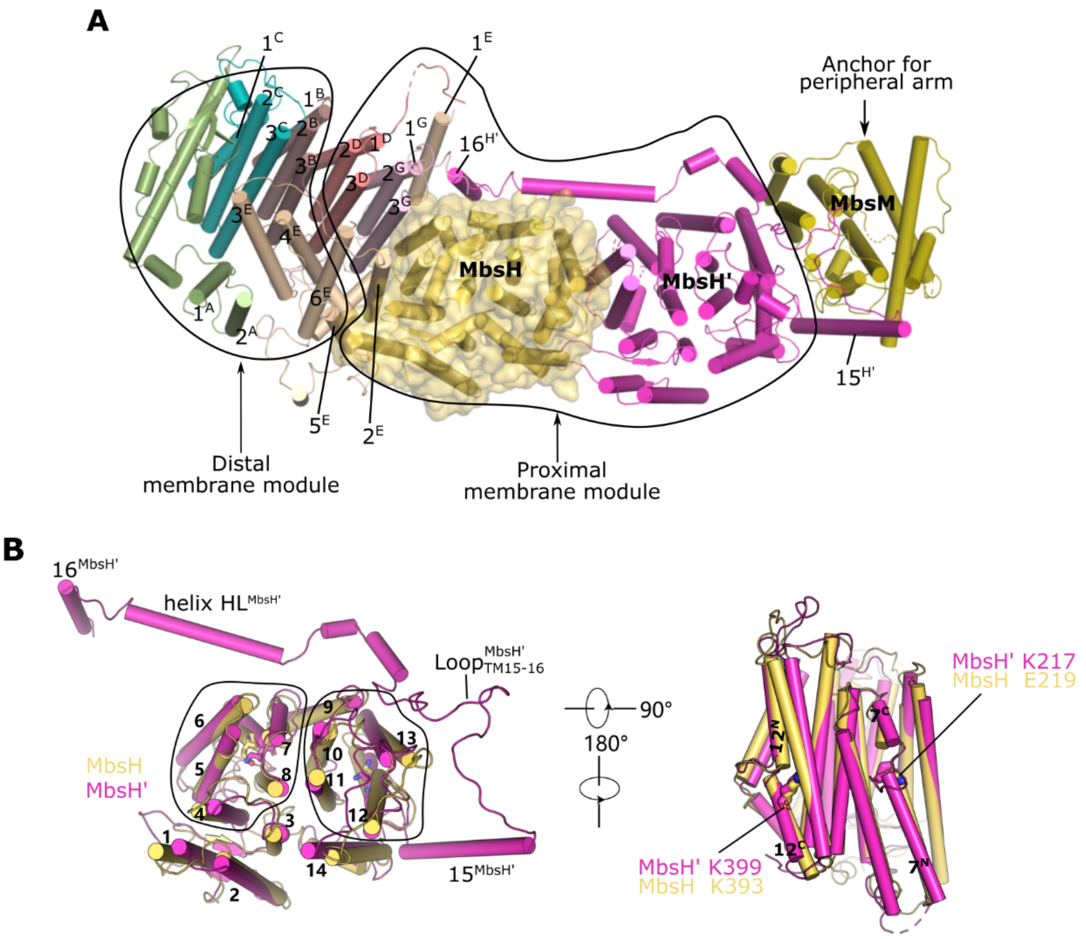

Supplementary Figure 3. Assembly of the membrane arm of MBS. (A) A top view of the membrane arm of the MBS monomer showing the arrangement of membrane subunits. The outlined proximal membrane module is composed of MbsD, MbsE TMH1-2, MbsG, MbsH and MbsH'. **(B)** Structural alignment of the antiporter-like subunits MbsH (yellow) and MbsH' (magenta) of the proximal membrane module. TM helices are numbered. Left panel, top view of the alignment viewed from cytosol. The two putative proton-translocating five-helix folding units are outlined. They are superimposable when one unit is turned upside down with respect to the other. Right panel, side view of the alignment from the membrane side (MbsH' TMH15-16 is hidden for clarity). Two discontinuous α-helices (TMH7 and TMH12) are labeled with key charged residues shown as spheres.

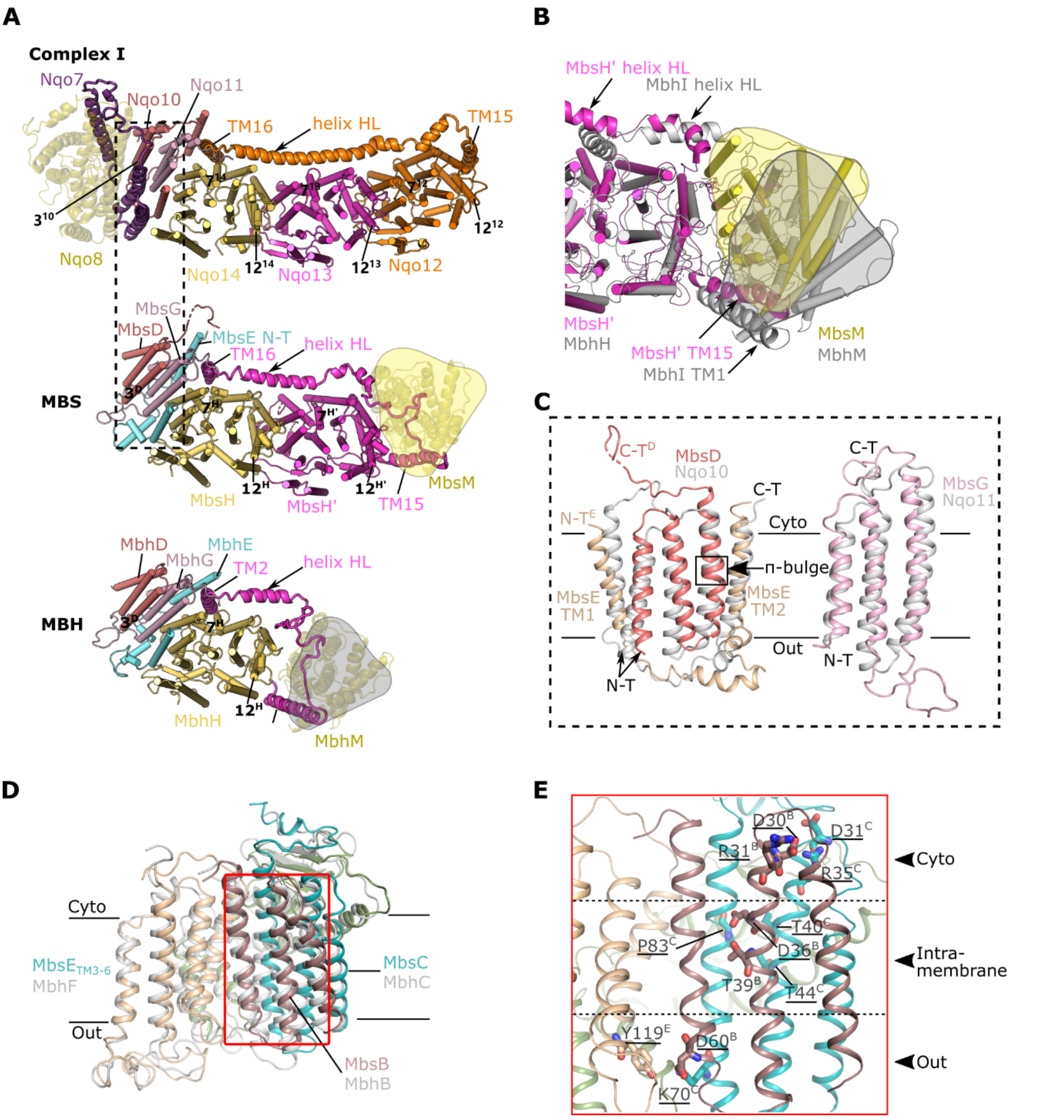

Supplementary Figure 4. Comparison of three different respiratory machineries: Complex I, MBH and MBS. (A) A structural overlay of the MBS proximal membrane module with its counterparts in Complex I and MBH. The alignment (as done in **Figure 3A**) is based on the respective proton-pumping antiporter-like subunits Nqo14, MbsH and MbhH of Complex I (*T. thermophilus*; PDB ID 4HEA), MBS and MBH (*P. furiosus*; PDB ID 6CFW). Complex I has 3 proton-pumping antiporter-like subunits in the proximal membrane module, MBS has 2, and MBH has one. The different sizes of the proximal modules are accommodated by different lengths of their HL helices. Complex I Nqo8, MBS MbsM and MBH MbhM (shown as transparent cartoons) serve as the membrane anchors for their respective hydrogenase modules. The peripheral module in Complex I is located at the opposite end of the membrane arm as compared to that of MBH and MBS. **(B)** Different membrane interfaces adjacent to the membrane-anchored peripheral module revealed by the alignment of their antiporter-like

subunits MbsH’ (MBS) and MbhH (MBH). Note the tight interface between MbsH’ and MbsM in MBS in contrast to the large gap between the two corresponding subunits in MBH. **(C)** A close-up view of the dashed box of the aligned structure in panel (**B**) shows the subunit-subunit correspondence between MBS and Complex I. **(D)** A close-up view of the shared Na^+-translocation module between MBH and MBS (as shown in **Fig. 3A**). (**E**) Putative Na^+-translocation path within the distal membrane module of MBS as outlined by the solid line red box in (**D**).

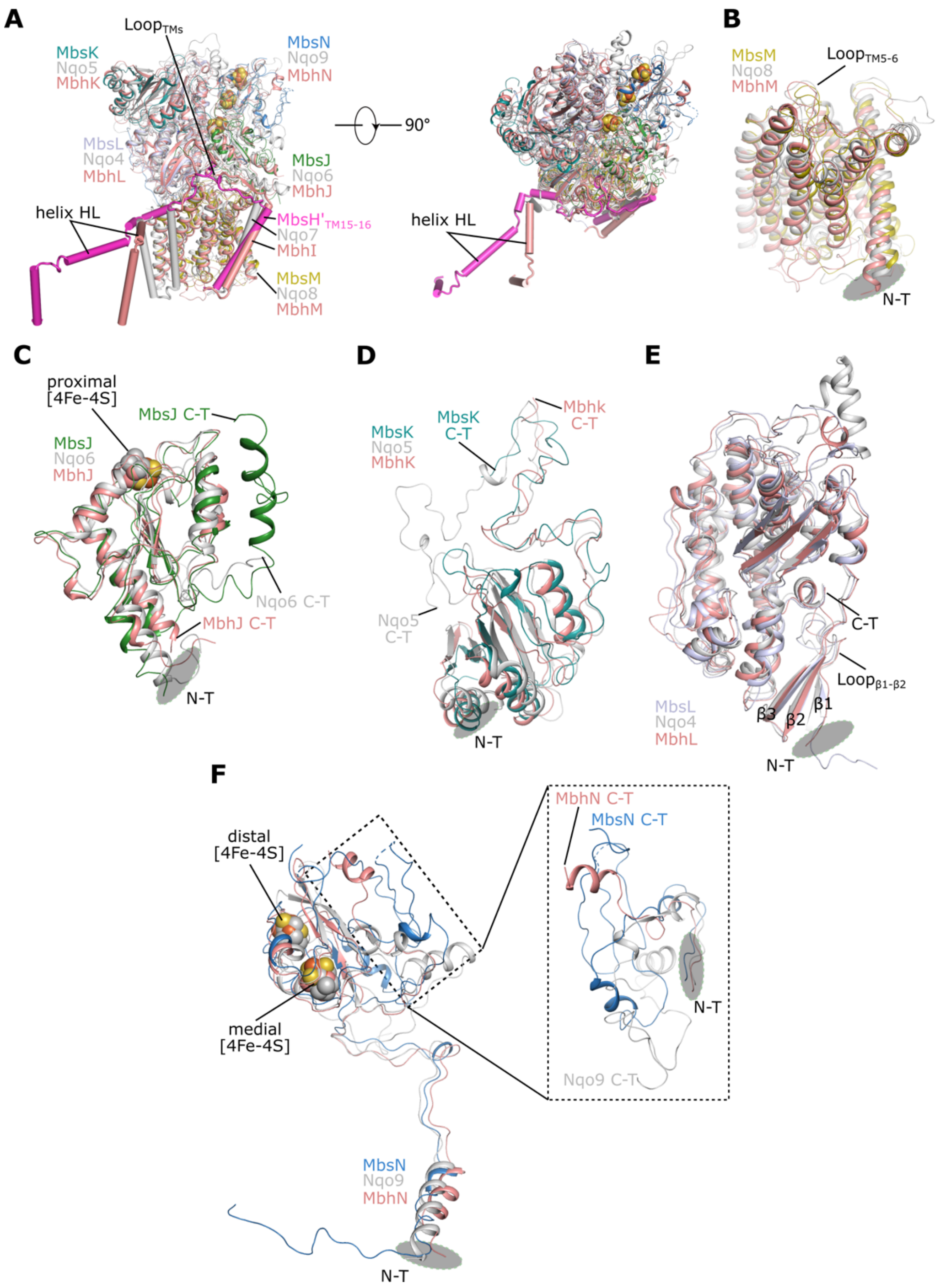

Supplementary Figure 5. A comparison of the membrane-anchored peripheral modules of MBS, MBH and Complex I. (A) Side (left) and top (right) views of the aligned membrane-anchored peripheral modules of MBS (colored as in **Figure 1C**), MBH (salmon) and Complex I (grey). Structural alignment is based on MBS MbsL, MBH MbhL, and Complex I Nqo4. **(B-F)** Zoomed views of the subunit-subunit correspondences based on the structural alignment in **A**. Their respective N-terminal ends (N-T) are marked by semi-transparent grey disks and the C-terminal ends by the text label “C-T”. (**B**) MbsM, MbhM and Nqo8. (**C**) MbsJ, MbhJ and Nqo6. (**D**) MbsK, MbhK and Nqo5. (**E**) MbsL, MbhL and Nqo4. (**F**) MbsN, MbhN and Nqo9.

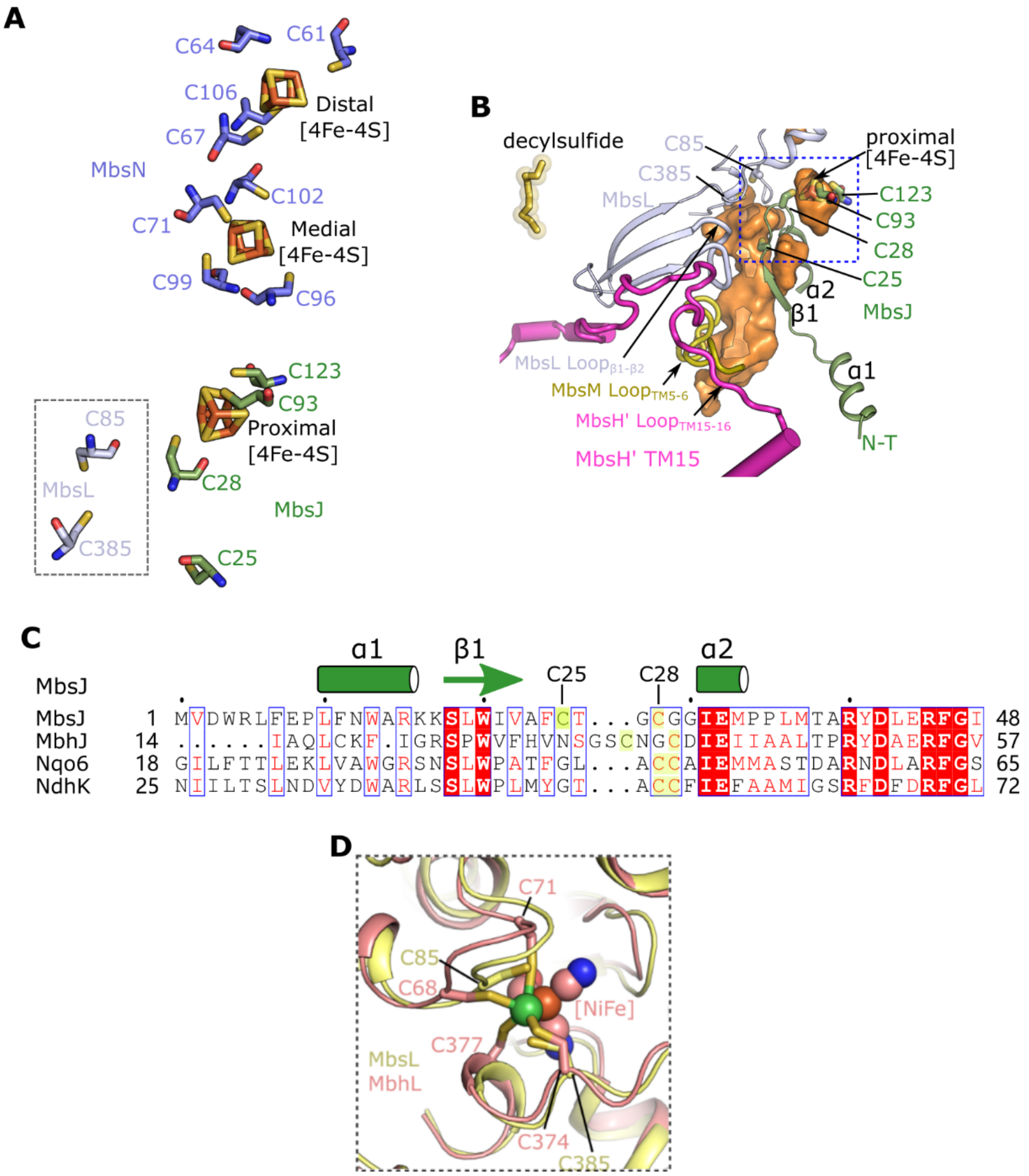

Supplementary Figure 6. Cysteine coordination of the [4Fe-4S] clusters in MBS. (A) Distribution of cysteine residues for the coordination of three [4Fe-4S] clusters. Note that MbsL C85 and C385 and MbsJ C25 near the proximal cluster don't participate in cluster interaction. **(B)** Near the proximal [4Fe-4S] cluster, a large chamber (shown as orange surface) is formed at the interface between the peripheral arm and membrane arm (as shown in **Figure 5A**), which defines the active site for the redox reaction. A possible substrate decylsulfide is shown in sticks and transparent spheres at the upper left corner, in the same scale as the proteins. MbsJ residues C28, C93 and C123 coordinate the proximal [4Fe-4S] cluster while a nearby free MbsJ C25 faces the chamber. Right below MbsJ C25, three interfacial loops (as highlighted in **Figure 6B**) define the boundaries of the chamber. **(C)** Sequence alignments of the structural motif (as shown in **B**) containing two variable cysteines (highlighted as yellow shade) for the coordination of proximal [4Fe-4S] cluster (C35 and C38 for MBH; C25 and C28

for MBS; C52 and C53 for NDH; C45 and C46 for Complex I). **(D)** MbsL C85 and C385 (as outlined by dashed box in panel **A**) correspond to two of the four [NiFe]-coordinating cysteines in MBH and its ancestor [NiFe] hydrogenase, as revealed by the structural comparison of MBS MbsL and MBH MbhL. The other two cysteines are lost, making a vestigial site unable for [NiFe] coordination in MBS.

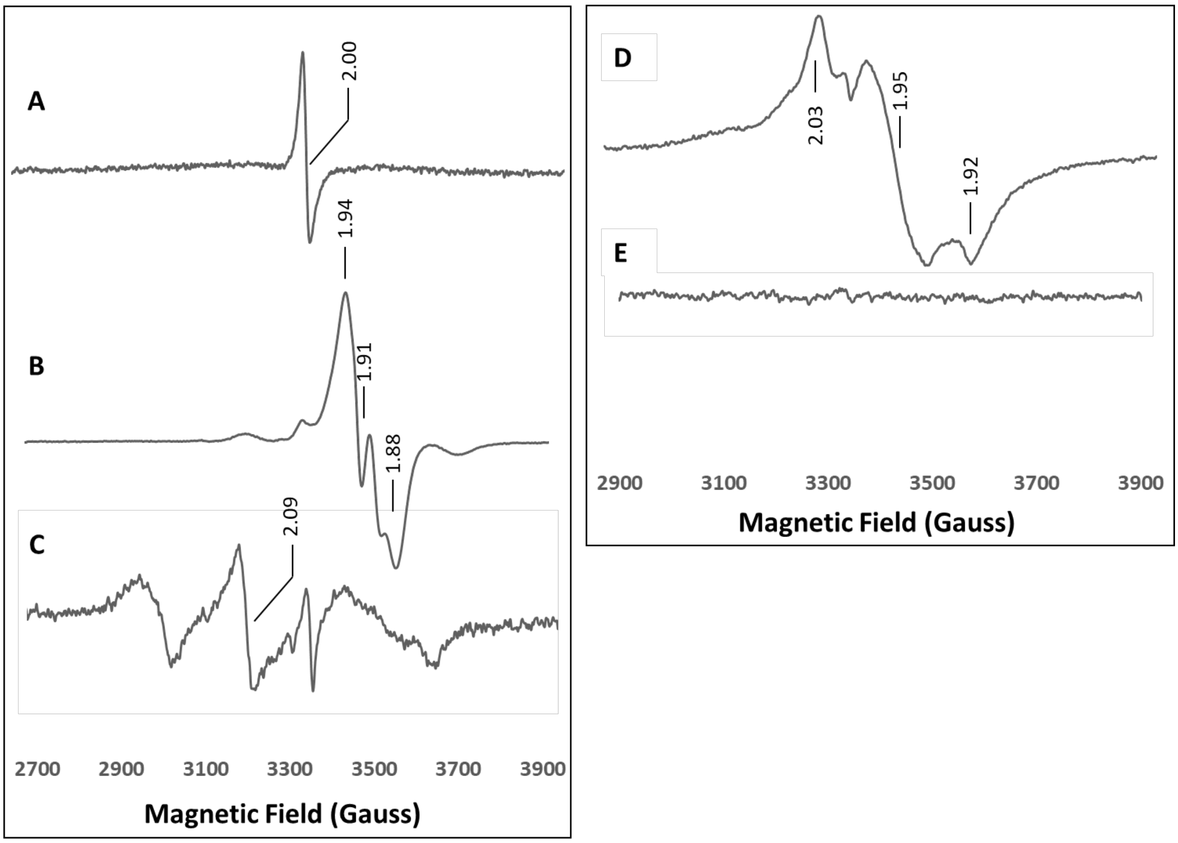

Supplementary Figure 7. EPR spectra of MBS and MBH. (A) EPR spectrum of dithionite - reduced MBS from S^0-grown cells showing a strong radical signal (g = 2.00). **(B)** EPR spectrum of dithionite-reduced MBS from non-S^0 grown cells. The g value (1.94) is characteristic of reduced [4Fe-4S] clusters. **(C)** EPR spectrum of FeCN-oxidized MBS from non-S^0 grown cells. The g value (2.09) is indicative of oxidized [3Fe-4S] clusters and corresponds to loss of the catalytic Fe atom upon oxidation. **(D)** EPR spectrum of dithionite-reduced MBH, indicating multiple reduced [4Fe-4S] clusters. **(E)** EPR spectrum of MBH after FeCN oxidation is silent, indicative of an oxidized [4Fe-4S] cluster. EPR parameters for each spectrum are temperature, 10K; microwave power, 20 mW; receiver gain, A: 75, B: 30, C: 300, D: 30, E: 30.
