# Supplementary material for: Structure of the respiratory MBS complex reveals iron-sulfur cluster catalyzed sulfane sulfur reduction in ancient life
Source: Nat Commun. 2020 Nov 23;11:5953. doi: 10.1038/s41467-020-19697-7 (PMC7684303; doi:10.1038/s41467-020-19697-7)
Supplement: Supplementary file 2 — Reporting summary [file 41467_2020_19697_MOESM2_ESM.pdf]

nature research

Corresponding author(s): Huilin Li
Michael Adams

Last updated by author(s): Oct 13, 2020

Reporting Summary

Nature Research wishes to improve the reproducibility of the work that we publish. This form provides structure for consistency and transparency in reporting. For further information on Nature Research policies, see our Editorial Policies and the Editorial Policy Checklist.

Statistics

For all statistical analyses, confirm that the following items are present in the figure legend, table legend, main text, or Methods section.

n/a	Confirmed	
☐	☒	The exact sample size (*n*) for each experimental group/condition, given as a discrete number and unit of measurement
☐	☒	A statement on whether measurements were taken from distinct samples or whether the same sample was measured repeatedly
☒	☐	The statistical test(s) used AND whether they are one- or two-sided<br>*Only common tests should be described solely by name; describe more complex techniques in the Methods section.*
☒	☐	A description of all covariates tested
☒	☐	A description of any assumptions or corrections, such as tests of normality and adjustment for multiple comparisons
☒	☐	A full description of the statistical parameters including central tendency (e.g. means) or other basic estimates (e.g. regression coefficient) AND variation (e.g. standard deviation) or associated estimates of uncertainty (e.g. confidence intervals)
☒	☐	For null hypothesis testing, the test statistic (e.g. *F*, *t*, *r*) with confidence intervals, effect sizes, degrees of freedom and *P* value noted<br>*Give P values as exact values whenever suitable.*
☒	☐	For Bayesian analysis, information on the choice of priors and Markov chain Monte Carlo settings
☒	☐	For hierarchical and complex designs, identification of the appropriate level for tests and full reporting of outcomes
☒	☐	Estimates of effect sizes (e.g. Cohen's *d*, Pearson's *r*), indicating how they were calculated

Our web collection on statistics for biologists contains articles on many of the points above.

Software and code

Policy information about availability of computer code

Data collection	Cryo-EM data collection used SerialEM 3.7beta in Titan Krios and EPU as implemented in Arctica by the manufacturer Thermo-Fisher Scientific. EPR spectra were recorded using a Bruker EMXplus CW EPR spectrometer controlled with a Bruker PremiumX Ultra low noise microwave bridge.
Data analysis	RELION 3.0, MotionCor2 1.1, CTFFIND4.1.10, cryoSPARC v2, ResMap 1.1.5, Coot 0.8.9, PHENIX 1.15, MolProbity 4.1, Chimera 1.8; PyMOL 1.8.

For manuscripts utilizing custom algorithms or software that are central to the research but not yet described in published literature, software must be made available to editors and reviewers. We strongly encourage code deposition in a community repository (e.g. GitHub). See the Nature Research guidelines for submitting code & software for further information.

Data

Policy information about availability of data

All manuscripts must include a data availability statement. This statement should provide the following information, where applicable:

- Accession codes, unique identifiers, or web links for publicly available datasets
- A list of figures that have associated raw data
- A description of any restrictions on data availability

The accession number for the atomic coordinates reported in this paper is PDB ID 6U8Y. The accession number for the EM density map reported in this paper is EMD-20692.

Field-specific reporting

Please select the one below that is the best fit for your research. If you are not sure, read the appropriate sections before making your selection.

- [x] Life sciences
- [ ] Behavioural & social sciences
- [ ] Ecological, evolutionary & environmental sciences

For a reference copy of the document with all sections, see nature.com/documents/nr-reporting-summary-flat.pdf

Life sciences study design

All studies must disclose on these points even when the disclosure is negative.

Sample size	We collected two datasets, one with 8352 and the other with 6355 raw movie micrographs in Titan Krios. After 2D classification, a total of 673,320 and 667,428 "good" particles that produced clear 2D class averages were retained for each of the two datasets. After 3D classification, best particles of the two datasets were pooled (230,673) for final refinement, leading to the final 3D map at 4.0 Å resolution.
Data exclusions	"Bad" raw particles that did not produce 2D class averages or 3D maps with defined features were excluded after 2D and 3D classifications.
Replication	Reproducibility resides in the large number of particles used to derive at the final 3D maps or 2D averages. The reliability and the resolution are measured by the gold-standard Fourier shell correlation. We also collected two independent datasets that both led to similar 3D maps. Replication efforts further included multiple refinement runs that all yielded similar 3D maps.
Randomization	The raw particles were randomly selected by computer program (RELIONS 3.0).
Blinding	The investigators were not blinded to the specific data points during data collection and analysis. Blinding was not feasible because our raw data are images that are visible, and in fact, visual inspection of the raw data is required as a part of quality control. However, the inclusion of the final data points in 3D structural calculation is determined by the program (RELIONS), not by the investigators manually.

Reporting for specific materials, systems and methods

We require information from authors about some types of materials, experimental systems and methods used in many studies. Here, indicate whether each material, system or method listed is relevant to your study. If you are not sure if a list item applies to your research, read the appropriate section before selecting a response.

Materials & experimental systems

n/a	Involved in the study	
☒	☐	Antibodies
☒	☐	Eukaryotic cell lines
☒	☐	Palaeontology and archaeology
☒	☐	Animals and other organisms
☒	☐	Human research participants
☒	☐	Clinical data
☒	☐	Dual use research of concern

Methods

n/a	Involved in the study	
☒	☐	ChIP-seq
☒	☐	Flow cytometry
☒	☐	MRI-based neuroimaging
